# Effect of an Intervention Package and Teamwork Training to Prevent Healthcare Personnel Self-contamination During Personal Protective Equipment Doffing

**DOI:** 10.1093/cid/ciz618

**Published:** 2019-09-13

**Authors:** Jennifer Andonian, Sadaf Kazi, Jennifer Therkorn, Lauren Benishek, Carrie Billman, Margaret Schiffhauer, Elaine Nowakowski, Patience Osei, Ayse P Gurses, Yea-Jen Hsu, David Drewry, Ellen R Forsyth, Arjun Vignesh, Ifeoluwa Oresanwo, Brian T Garibaldi, Kaitlin Rainwater-Lovett, Polly Trexler, Lisa L Maragakis

**Affiliations:** 1 Department of Hospital Epidemiology and Infection Control, Johns Hopkins Hospital; 2 Johns Hopkins Armstrong Institute for Patient Safety and Quality; 3 Department of Anesthesia and Critical Care Medicine, Johns Hopkins University School of Medicine; 4 Department of Applied Biological Sciences, Johns Hopkins Applied Physics Laboratory; 5 Department of Health Policy and Management, Johns Hopkins Bloomberg School of Public Health; 6 Department of Medicine, Johns Hopkins University School of Medicine; 7 Johns Hopkins Bloomberg School of Public Health, Department of Epidemiology, Baltimore, Maryland

**Keywords:** personal protective equipment doffing, self-contamination, inhalational exposure, viral hemorrhagic fever, biocontainment

## Abstract

**Background:**

More than 28 000 people were infected with Ebola virus during the 2014–2015 West African outbreak, resulting in more than 11 000 deaths. Better methods are needed to reduce the risk of self-contamination while doffing personal protective equipment (PPE) to prevent pathogen transmission.

**Methods:**

A set of interventions based on previously identified failure modes was designed to mitigate the risk of self- contamination during PPE doffing. These interventions were tested in a randomized controlled trial of 48 participants with no prior experience doffing enhanced PPE. Contamination was simulated using a fluorescent tracer slurry and fluorescent polystyrene latex spheres (PLSs). Self-contamination of scrubs and skin was measured using ultraviolet light visualization and swabbing followed by microscopy, respectively. Doffing sessions were videotaped and reviewed to score standardized teamwork behaviors.

**Results:**

Participants in the intervention group contaminated significantly fewer body sites than those in the control group (median [interquartile range], 6 [3–8] vs 11 [6–13], P = .002). The median contamination score was lower for the intervention group than the control group when measured by ultraviolet light visualization (23.15 vs 64.45, P = .004) and PLS swabbing (72.4 vs 144.8, P = .001). The mean teamwork score was greater in the intervention group (42.2 vs 27.5, P < .001).

**Conclusions:**

An intervention package addressing the PPE doffing task, tools, environment, and teamwork skills significantly reduced the amount of self-contamination by study participants. These elements can be incorporated into PPE guidance and training to reduce the risk of pathogen transmission.

## Teaser

An intervention package that addressed components of the task, tools, environment, and teamwork skills during personal protective equipment (PPE) doffing significantly reduced the amount of self-contamination by study participants. These elements can be incorporated into PPE guidance and training to prevent pathogen transmission.

More than 28 000 people were infected with Ebola virus during the 2014–2015 West African outbreak, resulting in more than 11 000 deaths [1]. Many of the deaths occurred among healthcare personnel whose infection risk was approximately 32% higher than that of the general population [1]. Enhanced personal protective equipment (PPE), which is used when caring for patients with high-consequence pathogens, consists of multiple components that require numerous steps and assistance to remove safely [2, 3]. The “doffing team” consists of the healthcare worker (HCW), a doffing assistant (DA) to help the HCW remove PPE components, and a trained observer (TO) to verbalize instructions and monitor the safety of the procedure [2]. This complex process presents a risk of self-contamination and pathogen transmission. In a recent study, it was found that hand or neck contamination during glove and gown removal was approximately 50% [4]. The amount of self-contamination may differ depending on the type of PPE and doffing technique, among other factors [5–8].

Teamwork is required to safely accomplish the PPE doffing steps. The importance of the TO and DA roles was highlighted in the Centers for Disease Control and Prevention (CDC) 2015 PPE guidelines [2]. Researchers have begun to explore team-centric and human factors engineering (HFE) principles and methods to improve infection prevention practices [3, 9–13]. These approaches provide information about possible routes of contamination and can help in the redesign of the doffing process to reduce errors [9]. Methods such as failure modes and effects analysis (FMEA) identify and prioritize risks in order to develop risk mitigation strategies [3, 9]. Teamwork skills and behaviors throughout the doffing process help to ensure that all steps are completed appropriately and any contamination is promptly identified and remediated.

**Figure 1.**
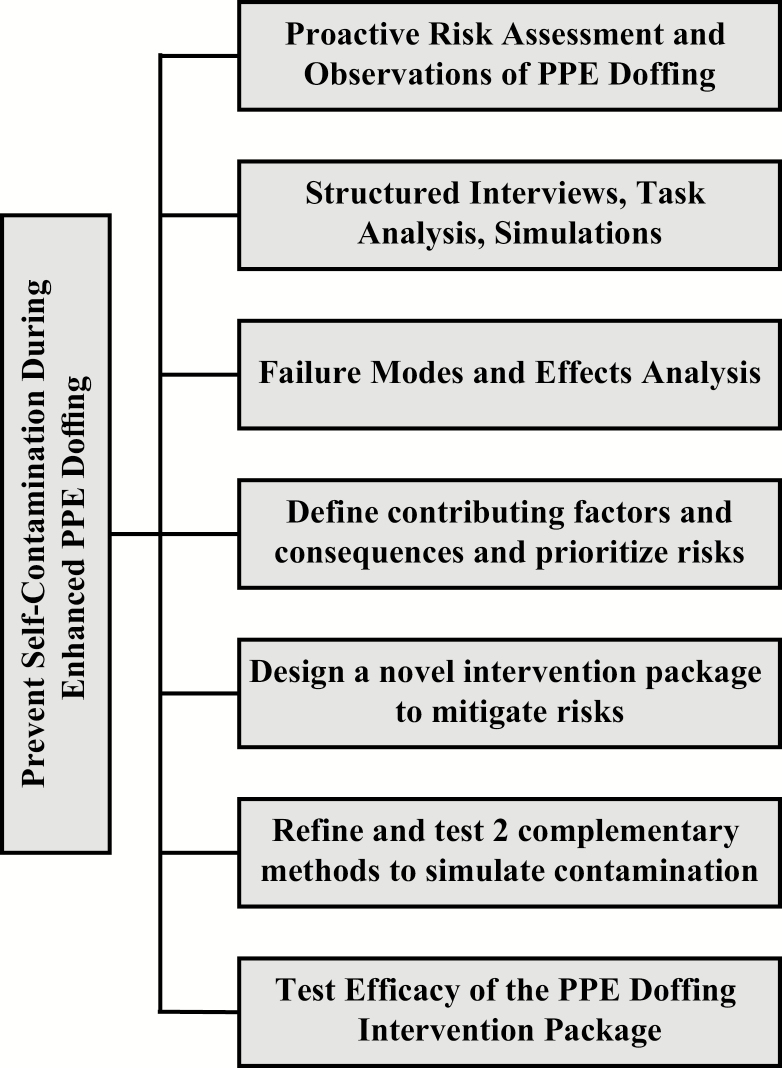
Components of the Johns Hopkins Prevention Epicenter studies to prevent self-contamination during enhanced personal protective equipment (PPE) doffing. The current report describes the final step of testing the efficacy of the PPE Doffing Intervention Package. Abbreviation: PPE, personal protective equipment.

The current study is part of a CDC-funded Prevention Epicenter project that uses HFE methods to analyze the PPE doffing process, determine risks and potential failure modes, design interventions to mitigate identified risks, and test the efficacy of the interventions to reduce the risk of self-contamination. Figure 1 illustrates the components of this project’s mixed methods approach that included contextual inquiry and hierarchical task analysis. The previously reported FMEA [3] identified 103 failure modes, of which the ones with the highest priority scores were walking between clean and dirty areas of the doffing space, failure to identify contamination and take steps to mitigate risk, failure to thoroughly disinfect all surfaces of gloves, and failure to complete adequate hand hygiene after each step of the doffing process. In the current study, mitigation strategies to address these prioritized risks were incorporated into a set of interventions that address the PPE doffing task, tools, environment, and teamwork skills. Our aim in this study was to test the efficacy of the intervention package in order to reduce the amount of self-contamination by study participants.

## METHODS

### Development of the Intervention Package

Previously identified and prioritized failure modes [3] and their associated contributing factors and consequences were used to develop a set of interventions to mitigate the risk of self-contamination during PPE doffing. Input was compiled from focus groups, semistructured interviews, and infection prevention and human factors experts. Interventions addressed various components of the doffing process, including tools/technology (eg, PPE selection), people (eg, roles, teamwork), task (eg, technical aspects of PPE removal), and environment (eg, doffing room characteristics) [14, 15]. Table 1 outlines the elements of the intervention package.

**Table 1.** Elements of an Intervention Package to Prevent Self-contamination During PPE Doffing.

Tools and Technology
Tape is applied to the top edge of the outer glove, securing it to the gown sleeve (tab for easier removal)
HCW wears thigh-high boot covers (rather than calf-high) taped to the scrubs at the top
HCW dons a pair of exam gloves for patient care (over the double gloves)
HCW dons an outer cover gown for patient care (with sleeves rather than an apron)
HCW dons a bouffant hair cover to contain hair before donning PAPR
Task
HCW, DA, TO examine personal protective equipment after donning each pair of gloves to ensure integrity
HCW doffs outer exam gloves and outer cover gown in the patient room before entering the doffing room
HCW shuffles feet on disinfectant soaked pad, then on dry pad prior to entering doffing space
DA opens doffing room door with disinfectant wipe for HCW to enter doffing room
During glove doffing, the first glove removed is discarded and not held in the other hand
HCW uses tab on tape at wrist to remove it from the glove/gown interface
DA folds back a 1- to 2-inch “cuff” on both edges of the gown after untying it and before the HCW removes it (to help ensure that the outer, contaminated portion does not touch the HCW during doffing)
DA removes tape from the top of the boot covers by pulling on the tab prior to assisting with boot removal process
Boot covers are removed at the end of the doffing process; legs are moved from dirty to clean side of the doffing space after each boot cover is removed
Handwashing with soap and water is required after leaving the doffing room
People
TO encourages closed-loop communication for HCW and DA
TO provides directions for each step with additional comments and reminders, as necessary, for adding support during the doffing process (eg, “remember to apply alcohol rub to all surfaces of the hands and fingers and all the way up to the wrists and tape”)
Teamwork skills of closed-loop communication, mutual support, situational awareness, and assertiveness to verbalize and express any safety concerns (eg, speak up/speak out) are emphasized for each of the doffing team members and included in the training curriculum
Environment
Mirrors on the wall of the patient room by the door to allow HCW to visually inspect for contamination and take remediation steps before entering the doffing space
Mirrors are present on both dirty and clean sides of the doffing room and incorporated into the doffing process to assist with visual inspection and spatial awareness
Floor of doffing room includes different colors to designate clean vs dirty areas; TO stays on the clean portion of the doffing space

Abbreviations: DA, doffing assistant; HCW, healthcare worker; PAPR, powered air-purifying respirator; TO, trained observer.

### Methods to Simulate PPE Contamination

Two PPE contamination simulation methods were developed and tested as reported previously [16]. Several fluorescent tracer slurries were tested for 3 criteria: even distribution on PPE, ability to transfer from PPE to scrubs and skin, and visualization on scrubs and skin for detection using ultraviolet light. The optimized fluorescent slurry consisted of fluorescent powder (Glitter Bug, Brevis Corporation, Salt Lake City, UT; 75 mg/mL) in a viscous suspension of grapeseed oil and water (1:6 oil-to-water ratio)] [16]. The fluorescent tracer mixture was applied to PPE using 1000 mL in a pesticide hand sprayer (RL Flo-Master, Lowell, MI; 2000 mL capacity) and 5 sweeping passes of sprayer from head to feet on the front and back of the HCW (Supplementary Material, Supplementary Figure 1). Detection was by direct visualization in a dark room using ultraviolet light. The number of body sites contaminated and the extent of contamination at each site were recorded.

The second method utilized fluorescent 2-µm polystyrene latex bead (PLSs) (G0200, Thermo Fisher Scientific, Waltham, MA) diluted in water. PLSs are commonly utilized in aerosol research and were used to simulate pathogens in another recent study that explored self-contamination while doffing PPE [17]. A PLS suspension (25 mL) was aerosolized using a 3-jet Collison nebulizer (Mesa Laboratories, Inc, Butler, NJ) for 4 minutes of continuous aerosol generation while the HCW turned 90° every 60 seconds. Sampling was conducted by swabbing hands, wrists, ears, and face with sterile foam-tipped swabs (Puritan Medical Products Co LLC, Guilford, ME) premoistened in filtered, deionized water with 0.05% Triton-X 100. PLSs were also sampled from air in the breathing zone using a Button Sampler (SKC Inc, Eighty Four, PA), operated at 4 L/min with 25-mm polytetrafluoroethylene filters of 3 µm pore size (SKC Inc). PLS detection was performed by counting via epifluorescent microscopy and quantifying the number PLSs per square centimeter of skin or per cubic meter of sampled air.

### Testing the PPE Doffing Intervention Package

The intervention package was tested in a randomized controlled trial in the Johns Hopkins Hospital Biocontainment Unit (JHH BCU) in Baltimore, Maryland, between 20 April 2018 and 4 May 2018. The institutional review board granted approval for the study. Forty-eight participants without prior experience doffing enhanced PPE were randomly assigned to the control or intervention condition and then to the role of HCW or DA. Study participants were blind to their group assignment. Infection preventionists (IPs) from the study team performed the role of the TO for all groups.

### Participant Training Process

Participants received approximately 2 hours of training prior to doffing PPE. The curriculum for both the treatment and control groups included a basic introduction to germ theory, modes of pathogen transmission, types and purpose of PPE, and basic tenets of infection prevention. Both control and intervention groups were shown the PPE components they would doff during the study.

Next, the control group participants watched a video that highlighted general facts about respiratory etiquette and the importance of covering your cough to prevent the spread of respiratory infections, followed by a video that demonstrated enhanced PPE doffing based on the 2015 CDC recommendations [2]. Study personnel annotated the video with a standardized script to prompt discussion on topics that included the doffing team roles, PPE components, importance of hand hygiene, and perceived risky doffing steps.

The intervention group participants watched a video about teamwork concepts and their application in healthcare. The training included information about potential risks in the doffing process, the benefit of teamwork in PPE doffing, and the roles and responsibilities of the doffing team members. Participants were instructed on teamwork strategies including use of verbal and nonverbal communication (eg, closed-loop communication); developing, maintaining, and updating situational awareness (eg, monitoring inadvertent contact of the HCW with other team members or room surfaces); mutually supporting team members; and the importance of verbalizing safety concerns. They were then shown a video that demonstrated the intervention package doffing process. As with the control group, study personnel annotated the video with a standardized script. Discussion points emphasized the nature of the teamwork interaction, strategies used to promote communication, situational awareness, the importance of hand hygiene, delineation of clean vs dirty spaces, and use of mirrors to assist in the doffing process.

### Study Procedures

After training, all participants were given the opportunity to ask questions and were informed about their randomly assigned role as either HCW or DA. They walked to the JHH BCU where they changed into paper scrubs and hospital footwear. Participants in the HCW role were fitted with a Button Aerosol sampler placed at the neck of the paper scrubs [16], provided wipes to remove make-up or oil from the face so as to not interfere with PLS detection, and examined with ultraviolet light in a darkened room to identify baseline fluorescence. Participants then proceeded to the donning room where study personnel helped them into their respective PPE ensembles (Table 2) using either the standard CDC ensemble checklist for the control group or an intervention group checklist.

**Table 2.** Personal Protective Equipment (PPE) Used by Control and Intervention Groups in a Study to Test Interventions to Reduce Self-contamination During PPE Doffing

Personal Protective Equipment Item	Product Name	Manufacturer	Use in Experiment
Gown	Cardinal Health SmartGown Breathable surgical gown	Cardinal Health	C, I
Gloves (outer)	Biogel Skinsense	Mölnlycke	C, I
Gloves (inner)	Biogel PI Indicator underglove	Mölnlycke	C, I
Isolation gown	MediChoice over-the-head poly coated gown	Owens and Minor	C,^a^ I
Boot covers	Hi Guard regular full coverage boot	Kimberly Clark	C
Boot covers	Sta-Dri nonskid boot leg	Sloan Corporation	I
PAPR	Air-Mate high-efficiency particulate air PAPR	3M	C, I
Tape	Duct tape	Office Depot	C, I
PAPR hood	Hood, white, tychem double shroud	3M	C, I
Exam gloves	PremierPro plus nitrile exam gloves	Medline	I

^a^The isolation gown sleeves were cut off to make an apron used over the gown for the control condition.

Abbreviation: C, control; I, intervention; PAPR, powered air-purifying respirator.

The HCWs proceeded to a designated room where their PPE was contaminated with fluorescent tracer slurry and PLS particles as described above. Participant teams then proceeded to designated rooms to begin the doffing procedures. The study design promoted unidirectional flow through the JHH BCU to prevent contamination of the space, participants, or study coordinators (Supplementary Material, Supplementary Figure 3). Study team members served as TOs for all teams. The TO for the control teams read the CDC guidelines checklist. The TO for the intervention teams read the intervention checklist, gave appropriate teamwork cues to encourage closed-loop communication, and talked through each step reminding the HCW and DA about details and risks (eg, “…make sure you get to all of the surfaces of the fingers and all the way up to the wrists when performing hand hygiene”). See Table 1 for the elements in the intervention condition that differed from the control condition.

### Teamwork Behaviors Scoring Process

Doffing sessions were videotaped, reviewed, and coded to assess teamwork dynamics using a task analysis of the process steps and substeps. Each step and substep were coded for communication, mutual support, and situational awareness. Each correctly performed substep received a score of 1 (Supplementary Material, Supplementary Tables 1 and 2).

### Contamination Reading and Data Collection Process

After doffing was complete, the HCWs were assessed for fluorescent tracer contamination with ultraviolet light in a darkened room. The number of body sites contaminated and the extent of contamination at each site were recorded on a standardized sheet (Supplementary Material, Supplementary Figure 2). The contamination forms were deidentified and assigned randomized numbers for scoring purposes. Two IPs, blinded to experimental assignment, independently scored each form using the contamination legend and then arrived at consensus scoring through discussion.

To sample for PLSs, swabs were obtained from preidentified areas on the HCW’s face, ears, wrists, and hands, and the filter was retrieved from the Button Sampler. The swabs and filters were processed to determine the number of PLSs present using methods described above and in Therkorn et al [16]. After doffing, each participant completed the National Aeronautics and Space Administration (NASA) Task Load Index (NASA-TLX) questionnaire to assess perceptions of workload during doffing and the Team Strategies and Tools to Enhance Performance and Patient Safety Teamwork Attitudes Questionnaire (T-TAQ) to assess attitudes toward teamwork [18, 19].

### Statistical Analyses

The median and interquartile range (IQR) were calculated for the number of body sites contaminated with fluorescent tracer and PLSs, respectively, and for quantitative scores for each type of simulated contamination. The fluorescent tracer contamination score was calculated by treating 1 dot as 1 point, 1 smear as 10 points, and 1 spray as 90 points. Summary scores were generated, including total PLSs, total fluorescent contamination scores from all body areas, and the count of body areas that were contaminated. A summary measure that combined PLS swab and florescent tracer results eliminated overlapping areas to avoid double counting. Wilcoxon rank sum tests were used to examine contamination and teamwork differences between the intervention and control groups; *t* tests were used to compare differences in workload and teamwork attitudes between the 2 groups. A *P* value of <.05 was considered to be statistically significant. All statistical analyses were performed using Stata 15.1 (Stata Corp, College Station, TX).

## RESULTS

Forty-eight study participants (35 females, 13 males) were randomly assigned to the control (n = 22) or intervention group (n = 26). None of the participants had prior experience doffing enhanced PPE. Participants in each study arm were randomly assigned to the role of control HCW (n = 11), control DA (n = 11), intervention HCW (n = 13), or intervention DA (n = 13). For the fluorescent tracer, 11 HCWs (84.6%) in the intervention group and all 13 control HCWs (100%) contaminated at least 1 body area. All HCWs in both groups contaminated at least 1 body area with PLSs. HCW self-contamination, as measured by a composite number of body sites with any contamination from either the fluorescent tracer or PLSs, was significantly lower in the intervention group than the control group (median [IQR] 6 [3–8] vs 11 [6–13], *P* = .002; see Table 3]. Self-contamination was significantly lower in the intervention group compared with the control group when measured by number of body sites contaminated by fluorescent tracer, number of body sites contaminated by PLSs, cumulative fluorescent tracer score, or cumulative PLS score (Table 3). The airborne concentration of PLSs recovered from the Button Aerosol samplers did not differ significantly between groups.

**Table 3.** Self-contamination of Scrubs and Skin During a Trial of Personal Protective Equipment Doffing

	Median (Interquartile Range)		*P* Value^a^
Self-contamination Type and Location	Intervention (n = 13)	Control (n = 11)	
Count of contaminated sites (combination of fluorescent tracer and PLS sites contaminated)^b^	6 (3–8)	11 (6–13)	**.002**
Fluorescent tracer surface contamination			
Number of contaminated sites	1 (1–2)	5 (2–5)	**.003**
Sum of contamination scores^c^	2 (1–5)	13 (9–141)	**.004**
Head	0 (0–0)	0 (0–0)	…
Neck	0 (0–0)	0 (0–0)	.277
Right shoulder	0 (0–0)	0 (0–0)	…
Left shoulder	0 (0–0)	0 (0–0)	.116
Mid-torso front	0 (0–0)	0 (0–1)	**.049**
Mid-torso back	0 (0–0)	0 (0–0)	…
Waist front	0 (0–0)	0 (0–0)	.450
Waist back	0 (0–0)	0 (0–0)	…
Right arm (including elbows)	0 (0–0)	0 (0–2)	**.020**
Left arm (including elbows)	0 (0–0)	0 (0–0)	…
Wrists front	0 (0–0)	0 (0–0)	…
Wrists back	0 (0–0)	0 (0–0)	.277
Hands front	0 (0–0)	0 (0–0)	.116
Hands back	0 (0–0)	0 (0–0)	.277
Thighs front	0 (0–2)	0 (0–2)	.728
Thighs back	0 (0–0)	3 (0–10)	**.008**
Knees front	0 (0–0)	0 (0–1)	.253
Knees back	0 (0–0)	0 (0–10)	.094
Lower leg front	0 (0–0)	0 (0–4)	.348
Lower leg back	0 (0–0)	3 (0–9)	**.035**
Ankles front	0 (0–0)	0 (0–1)	.085
Ankles back	0 (0–0)	0 (0–0)	.904
PLS surface contamination			
Number of contaminated sites	4 (2–5)	5 (5–8)	**.020**
Sum of contamination scores (number of PLS by microscopy)	72.4 (36.2–96.5)	144.8 (108.6–241.3)	**.001**
Forehead	0 (0–12.1)	0 (0–12.1)	.835
Left ear	0 (0–12.1)	0 (0–24.1)	.472
Right ear	0 (0–12.1)	0 (0–24.1)	.578
Chin	0 (0–0)	0 (0–24.1)	**.027**
Left cheek	0 (0–12.1)	0 (0–12.1)	.808
Right cheek	0 (0–12.1)	0 (0–12.1)	.834
Left inner wrist	12.1 (0–24.1)	12.1 (0–12.1)	.927
Right inner wrist	12.1 (0–12.1)	12.1 (0–36.2)	.261
Left back of hand	0 (0–0)	24.1 (12.1–36.2)	**<.001**
Right back of hand	0 (0–12.1)	12.1 (0–24.1)	.115
Left forefinger to thumb	0 (0–0)	12.1 (0–12.1)	.201
Right forefinger to thumb	0 (0–0)	12.1 (0–48.3)	**.025**
Estimated airborne concentration of PLS (number per cubic centimeter)	68.2 (17.4–329.8)	63.8 (0–168.9)	.599

The bold text indicates P values that are statistically significant at a value of P < .05.

Abbreviations: PLS, polystyrene latex sphere.

^a^From Wilcoxon rank sum tests.

^b^Fluorescent tracer contamination of head and hands is not included in the summary metric to avoid double counting with the PLS surface contamination.

^c^Contamination score is derived from the contamination categorized into dot, smear, and spray. One dot is 1 point; smear is scored as the maximum dots in a body area; and spray is scored as the maximum score of smear in a body area.

Coding and scoring of teamwork behaviors exhibited in the videotaped doffing sessions were completed for 10 intervention and 11 control teams. Technical difficulties resulted in missing videotapes for 3 intervention teams. Overall, intervention teams demonstrated significantly more teamwork behaviors during risky doffing steps (median [IQR] 27.1 [22.9–34.3]) than control teams (9.1 [6.3–14.7], *P *< .001). Intervention teams completed more behaviors associated with situational awareness (*M*_intervention_ = 24.9 [20.6–28.4] vs *M*_control_ = 18.1 [17.1–24.7], *P *< .05) and closed-loop communication (*M*_intervention_ = 24.5 [20.3–26.8] vs *M*_control_ = 9.4 [7.5–16.3], *P *< .01). Table 4 displays descriptive and inferential statistics on teamwork doffing behaviors. Both control and intervention groups reported positive teamwork attitudes. The intervention group had significantly higher positive attitudes about team structure compared with the control group (*t* [46] = 1.86, *P* = .04, 1-tailed test; *M*_intervention_ = 4.6, *M*_control_ = 4.4). No other subscales of the T-TAQ were significantly different between the control and intervention groups. According to the NASA-TLX questionnaire, participants in the intervention group perceived marginally higher mental demand compared to those in the control group (*t* [46] = 1.61, *P* = .055, 1-tailed test; *M*_intervention_ = 52.57, *M*_control_ = 41.92).

**Table 4.** Descriptive and Inferential Statistics for Teamwork Doffing Behaviors and Competencies

Teamwork Steps, Roles, and Competencies	Intervention (n = 10 Groups)			Control (n = 11 Groups)			
		Percent Behavior Compliance			Percent Behavior Compliance		*P* Value
	No. of Behaviors	Median	IQR	No. of Behaviors	Median	IQR	
All teamwork steps	175	25.1	21.0–28.6	130	17.5	14.2–20.0	.001
High-risk teamwork steps	35	27.1	22.9–34.3	35	9.1	6.3–14.7	<.001
Healthcare worker role	99	14.6	11.1–16.7	73	11.0	9.7–11.9	.062
Doffing assistant role	76	34.9	32.4–43.4	57	22.8	20.4–28.1	.001
Situational awareness	102	24.9	20.6–28.4	74	18.1	17.1–24.7	.020
Communication	71	24.5	20.3–26.8	53	9.4	7.5–16.3	.002
Mutual support	2	100.0	50.0–100.0	3	66.7	33.3–100.0	.103

Abbreviation: IQR, interquartile range.

## DISCUSSION

Self-contamination has been shown to occur frequently during PPE doffing and poses a risk of pathogen transmission that can be deadly in the case of Ebola and other high-consequence pathogens [20, 21]. As in other PPE doffing studies, we found that HCWs self-contaminated while doffing enhanced PPE and that PPE ensemble choice and environmental features may impact the risk of contamination [6, 20–22]. The study also shows that an intervention package designed to mitigate high-priority risks of the PPE doffing process can significantly reduce the amount of self-contamination that occurs. Self-contamination in this study was significantly lower in the intervention group compared with the control group whether measured by fluorescent tracer or PLSs and whether analyzed by the number of body sites contaminated or by summary scores of either metric or both.

The components of the intervention package in this study target high-risk doffing steps and address various elements of the doffing task, tools/technology, people, and environment. Although it is impossible to tell how much each element of the intervention package contributed to the overall risk reduction, simple changes, such as a different type of boot cover or adding an isolation gown and examination gloves as an additional protective layer, may have contributed to lowering the self-contamination risk. Elements of the doffing task itself, such as modification of the glove removal protocol so that the first removed glove is discarded rather than being held or folding back the edges of the gown to help the HCW avoid contaminated surfaces, may have also contributed to the reduced risk. Similarly, elements of the doffing environment, including clear demarcation of clean vs dirty areas of the doffing space and the inclusion of mirrors to enhance visual recognition of contamination and spatial awareness, may reduce risk.

According to the present study’s results, teamwork skills appear to be an important aspect of doffing enhanced PPE safely. The 2015 CDC guidance calls for both a DA and a TO to assist the HCW. This study’s intervention took this one step further, defining specific roles and responsibilities and training the participants to practice core teamwork skills such as closed-loop communication, mutual support, situational awareness, and speaking up about safety concerns. Indeed, the results of the study show that the intervention group not only demonstrated significantly more teamwork behaviors but also reported the doffing process to be easier than did the control group. The role of the TO was a central component of the intervention. The TOs in the intervention group utilized verbal prompts that appeared to promote teamwork behaviors. It is unclear whether teamwork training, TO behaviors, or both led to increased teamwork in the intervention group. These results suggest, however, that a knowledgeable TO who engages in active promotion of safe practices and teamwork is an important individual who can be influential in guiding the team members and keeping them safe.

In this study, we found that the 2 methods of simulated contamination, fluorescent tracer and PLSs, are complementary when detecting self-contamination events. Each has strengths and limitations, but together they allow a more complete assessment of the extent of self-contamination. Fluorescent tracer slurry facilitates detection of contamination on scrubs or other fabric worn underneath the PPE. The PLSs, on the other hand, enabled detection of skin contamination that could not be detected by the fluorescent tracer slurry alone. PLSs are more sensitive than fluorescent tracer in detecting contamination, but the samples are also more difficult, expensive, and time-consuming to process.

The presence of PLS contamination in the air samples from the Button Sampler is a provocative yet inconclusive finding. There was no significant difference between the control and intervention groups, but contamination was seen in both, raising the possibility that infectious particles might reaerosolize and pose an inhalational infectious risk during PPE doffing. PLSs and fluorescent slurry cannot, however, represent the relative risk of infection since they do not indicate viability and pathogens may be more or less likely to reaerosolize than PLSs. More research is needed to answer the questions raised by these preliminary data.

This study has limitations including the fact that the methods of simulated contamination do not directly represent viable organisms and may over- or underestimate the risk of pathogen transmission. It is a single-centered study that tested 1 PPE ensemble and may not be generalizable to all situations. There are some differences between the prioritized high-risk failure modes identified in this study and those identified by another group of authors whose work is reported in this supplement [23]. This may be due to variation in individual perceptions of the experts and participants in the focus groups who were interviewed for the 2 studies. The risk of self-contamination among novice participants in this study may not be representative of the risk to more experienced personnel.

Nevertheless, this study shows that an intervention package that addresses components of the task, tools, environment, and teamwork during PPE doffing significantly reduced the amount of self-contamination by study participants. These elements can be incorporated into enhanced PPE guidance and training to prevent pathogen transmission. The methods developed in this study may also be used to investigate self-contamination during doffing of “regular” PPE and to optimize PPE doffing training and protocols.

## Supplementary Data

Supplementary materials are available at *Clinical Infectious Diseases* online. Consisting of data provided by the authors to benefit the reader, the posted materials are not copyedited and are the sole responsibility of the authors, so questions or comments should be addressed to the corresponding author.

ciz618_suppl_Supplementary_Information

## Notes

***Acknowledgments.*** The authors thank contributing members of the Johns Hopkins Epicenter Team for their assistance in this project and all of our volunteer participants.

***Financial support.*** This work was supported by funding from the Centers for Disease Control and Prevention–Prevention Epicenters Program (cooperative agreement 1U54CK000447).

***Supplement sponsorship. This article appears as part of the supplement “Personal Protective Equipment for Preventing Contact Transmission of Pathogens: Innovations from CDC’s Prevention Epicenters Program,” sponsored by the CDC’s Prevention Epicenters Program.***

***Potential conflicts of interest.*** D. D. reports having a patent pending for a method for release and tracking of aerosol particles within a hospital environment. K. R. L. reports having a patent pending for a method for release and tracking of aerosol particles within a hospital environment. All other authors report no potential conflicts of interest. All authors have submitted the ICMJE Form for Disclosure of Potential Conflicts of Interest. Conflicts that the editors consider relevant to the content of the manuscript have been disclosed.
