# Supplementary material for: Effect of an Intervention Package and Teamwork Training to Prevent Healthcare Personnel Self-contamination During Personal Protective Equipment Doffing
Source: Clin Infect Dis. 2019 Sep 13;69(Suppl 3):S248–55. doi: 10.1093/cid/ciz618 (PMC6761361; doi:10.1093/cid/ciz618)
Supplement: ciz618_suppl_Supplementary_Information [file ciz618_suppl_supplementary_information.docx]

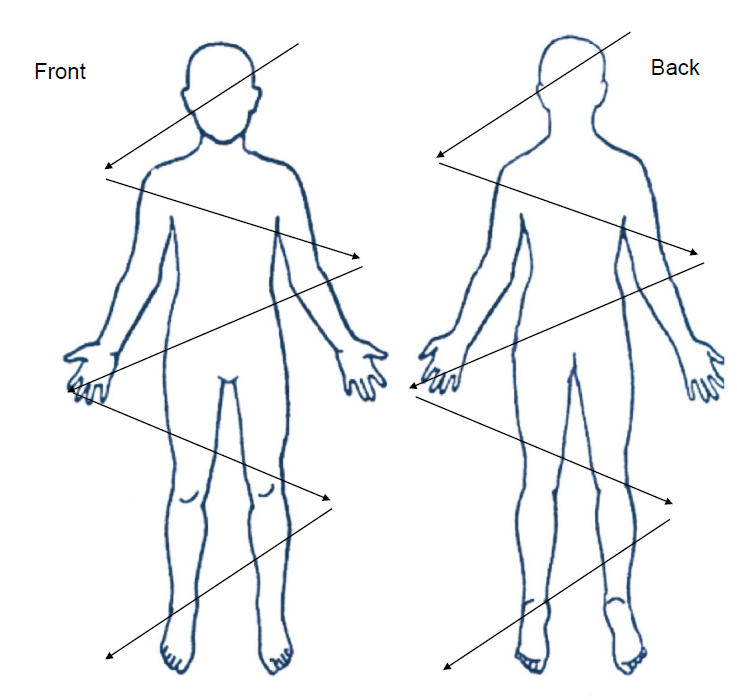


**Figure S1**. Sweeping method for contamination of participants using fluorescent tracer slurry.

**Contamination procedure:**

1. Have participant face towards you with palms facing forward

2. Shake spray bottle to mix the solution

3. Pump the spray bottle 5 times using the handle

4. Ensure you are 2 feet away from the participant

5. Start at the head of the subject and pull the nozzle to initiate spraying

6. Sweep the bottle right to left, then left to right working downwards towards the subjects’ feet

7. Ask participant to turn 180 degrees

8. Follow steps 2-6 to contaminate the back of the participant


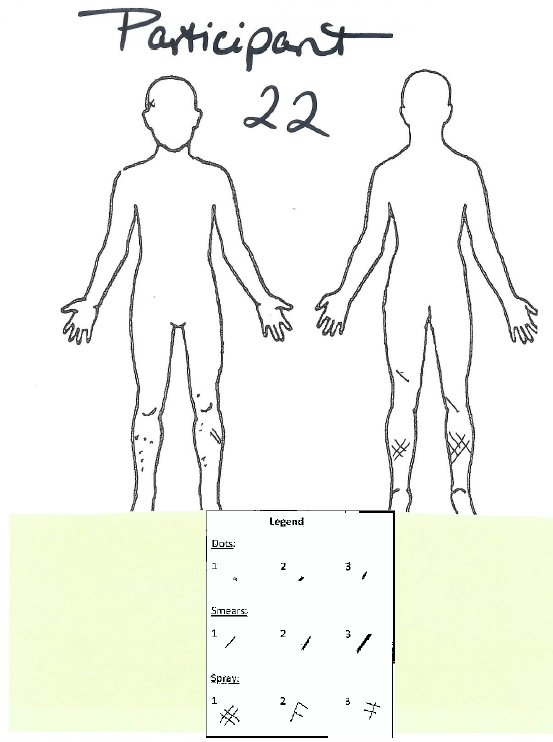


**Figure S2. Example of a de-identified self-contamination form for scoring the amount of fluorescent tracer slurry seen on scrubs and skin when visualized by black light inspection in a study of self-contamination after personal protective equipment doffing.** The location of contamination was recorded on the figure and either dots, lines, or hash marks were used to indicate the relative amount of contamination as dots, smears, or larger areas of sprays.


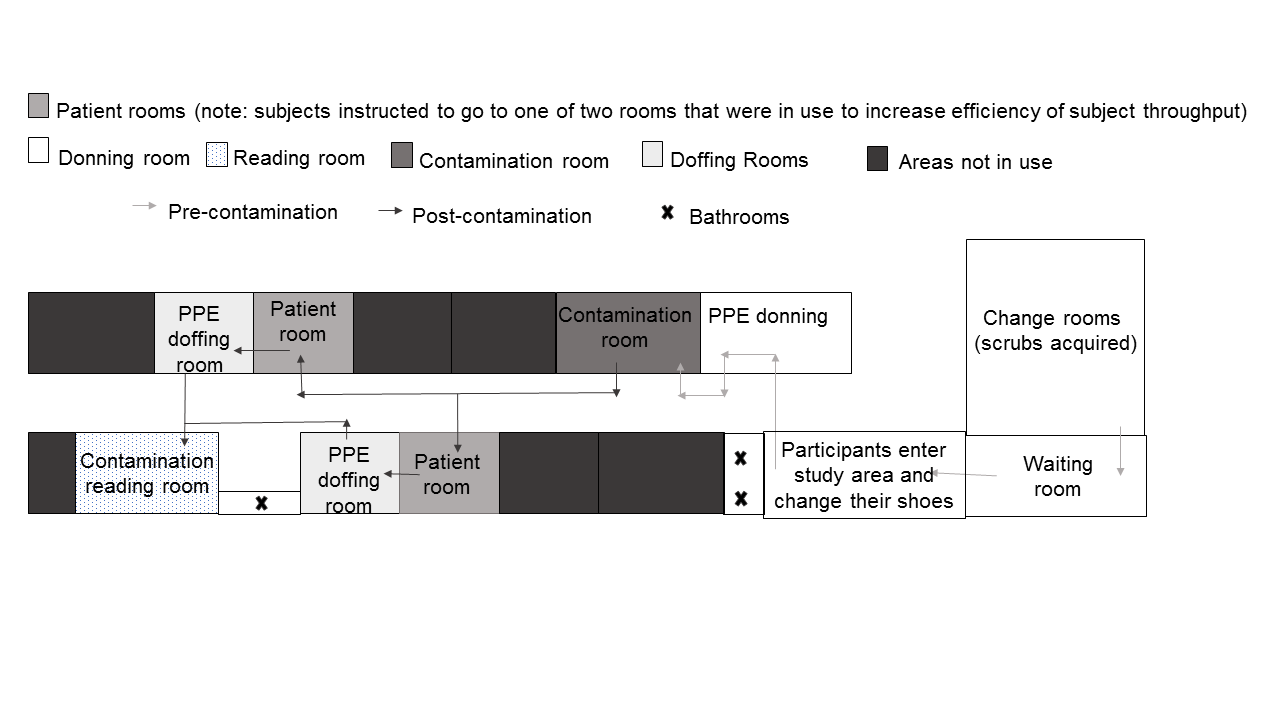
**Figure S3. Participant directional flow through the biocontainment unit (BCU) during a study of self-contamination during personal protective equipment (PPE) doffing**.

| **Table S1. Observation Tool: Control Order** | | | | | |
| --- | --- | --- | --- | --- | --- |
| **STEP** | **ROOM  1 = Patient 2 = Doffing** | **COMPETENCY** | **ACTOR 1 = HCW 2 = Buddy 3 = TO** | **BEHAVIOR** | **0 = N 1 = Y 2 = Uncertain 3 = NA OBSERVED** |
| **Step 1: PREPARATION FOR DOFFING (in patient room)** | | | |  |  |
| C1.01 | 1 | COM | 1 | **HCW** signals (vbl and/or nvbl) to engage TO and Buddy |  |
| C1.02 | 1 | COM | 3 | **TO** acknowledges HCW (vbl and/or nvbl) |  |
| C1.03 | 1 | SA | 3 | **TO** asks team if they are ready to exit room (proceed to next step) |  |
| C1.04 | 1 | COM | 1 | **HCW** indicates readiness to exit |  |
| C1Addtl | 1 |  |  | **Additional TMWK Behaviors observed in Step 1** |  |
| **Step 2: EXITING PATIENT ROOM; ENTERING DOFFING ROOM** | | | | |  |
| C2.01 | 2 | MS | 3 | **TO** verbally describes act of entering doffing room |  |
| C2.02 | 2 | MS | 3 | **TO** mimes act of entry |  |
| C2.03 | 2 | SA | 3 | **TO** watches HCW enter doffing room |  |
| C2.04 | 2 | SA | 2 | **Buddy** watches HCW enter doffing room |  |
| C2.05 | 2 | SA | 3 | **TO** introduces self and Buddy to HCW |  |
| C2.06 | 2 | SA | 3 | **TO** describes roles and responsibilities of all team members |  |
| C2.07 | 2 | SA | 3 | **TO** orients HCW to the doffing room layout |  |
| C2.08 | 2 | SA | 3 | **TO** emphasizes importance of maintaining spatial distance from trashcan and other surfaces |  |
| C2.09 | 2 | MS | 3 | **TO** encourages all team members to speak up and/or stop the doffing process when they have a concern |  |
| C2.10 | 2 | COM | 1 | **HCW** signals (vbl and/or nvbl) agreement with rules |  |
| C2.11 | 2 | COM | 2 | **Buddy** signals (vbl and/or nvbl) agreement with rules |  |
| C2.12 | 2 | SA | 3 | **TO** points out places of possible contamination to team or indicates that none is observed |  |
| C2.13 | 2 | SA | 1 | **HCW** points out places of possible contamination to team or indicates that none is observed |  |
| C2.14 | 2 | SA | 2 | **Buddy** points out places of possible contamination to team or indicates that none is observed |  |
| C2.15 | 2 | SA | 3 | **TO** asks team if they are ready to proceed to next step |  |
| C2.16 | 2 | COM | 2 | **Buddy** indicates readiness to move on |  |
| C2.17 | 2 | COM | 1 | **HCW** indicates readiness to move on |  |
| C2Addtl | 2 |  |  | **Additional TMWK Behaviors observed in Step 2** |  |
| **Step 3: ASSESS HCW PPE FOR VISIBLE CONTAMINATION (in doffing room)** | | | | |  |
| C3.01 | 2 | COM | 1 | **HCW** signals (vbl and/or nvbl) understanding of TO instructions or asks for clarification. |  |
| C3.02 | 2 | SA | 3 | **TO** visually inspects HCW for visible contamination. |  |
| C3.03 | 2 | SA | 2 | **Buddy** visually inspects HCW for visible contamination |  |
| C3.04 | 2 | MS | 1 | **HCW** turns in circles to facilitate Buddy and TO assessment of PPE for contamination |  |
| C3.05 | 2 | SA | 1 | **HCW** points out places of possible contamination to team or indicates that none is observed |  |
| C3.06 | 2 | SA | 2 | **Buddy** points out places of possible contamination to team or indicates that none is observed |  |
| C3.07 | 2 | SA | 3 | **TO** points out places of possible contamination to team or indicates that none is observed |  |
| C3.08 | 2 | SA | 3 | **TO** asks team if they are ready to proceed to next step |  |
| C3.09 | 2 | COM | 2 | **Buddy** indicates readiness to move on |  |
| C3.10 | 2 | COM | 1 | **HCW** indicates readiness to move on |  |
| C3Addtl | 2 |  |  | **Additional TMWK Behaviors observed in Step 3** |  |
| **Step 4: HAND HYGIENE PERFORMANCE #1 (HCW only, in doffing room)** | | | | |  |
| C4.01 | 2 | MS | 3 | **TO** verbally describes act of HH |  |
| C4.02 | 2 | MS | 3 | **TO** mimes act of HH |  |
| C4.03 | 2 | COM | 1 | **HCW** signals (vbl and/or nvbl) understanding of TO instructions or asks for clarification. |  |
| C4.04 | 2 | SA | 1 | **HCW** ‘talks out’ the HH process |  |
| C4.05 | 2 | SA | 2 | **Buddy** watches HCW perform HH |  |
| C4.06 | 2 | SA | 3 | **TO** watches HCW perform HH |  |
| C4.07 | 2 | MS | 3 | **TO** offers reinforcing and/or corrective feedback on HCW’s HH |  |
| C4.08 | 2 | SA | 1 | **HCW** signals (vbl and/or nvbl) when complete |  |
| C4Addtl | 2 |  |  | **Additional TMWK Behaviors observed in Step 4** |  |
| **Step 5: YELLOW ISOLATION GOWN REMOVAL** | | | |  |  |
| C5.01 | 2 | MS | 3 | **TO** verbally describes act of isolation gown removal. |  |
| C5.02 | 2 | MS | 3 | **TO** mimes act of isolation gown removal. |  |
| C5.03 | 2 | COM | 1 | **HCW** signals (vbl and/or nvbl) understanding of instructions or asks for clarification |  |
| C5.04 | 2 | SA | 1 | **HCW** ‘talks out’ the gown removal process |  |
| C5.05 | 2 | SA | 2 | **Buddy** watches HCW remove gown |  |
| C5.06 | 2 | SA | 3 | **TO** watches HCW remove gown |  |
| C5.07 | 2 | MS | 3 | **TO** offers HCW reinforcing and/or corrective feedback on gown removal |  |
| C5.08 | 2 | SA | 1 | **HCW** signals (vbl and/or nvbl) when complete |  |
| C5.09 | 2 | SA | 3 | **TO** asks team if they are ready to proceed to next step |  |
| C5.10 | 2 | COM | 2 | **Buddy** indicates readiness to move on |  |
| C5.11 | 2 | COM | 1 | **HCW** indicates readiness to move on |  |
| C5Addtl | 2 |  |  | **Additional TMWK Behaviors observed in Step 5** |  |
| **Step 6: ASSESS FOR VISIBLE CONTAMINATION** | | | |  |  |
| C6.01 | 2 | COM | 1 | **HCW** signals (vbl and/or nvbl) understanding of TO instructions or asks for clarification. |  |
| C6.02 | 2 | MS | 1 | **HCW** turns in circles to facilitate Buddy and TO assessment of PPE for contamination |  |
| C6.03 | 2 | SA | 2 | **Buddy** looks for contamination |  |
| C6.04 | 2 | SA | 3 | **TO** looks for contamination |  |
| C6.05 | 2 | SA | 1 | **HCW** points out places of possible contamination to team or indicates that none is observed |  |
| C6.06 | 2 | SA | 2 | **Buddy** points out places of possible contamination to team or indicates that none is observed |  |
| C6.07 | 2 | SA | 3 | **TO** points out places of possible contamination to team or indicates that none is observed |  |
| C6.08 | 2 | SA | 3 | **TO** asks team if they are ready to proceed to next step |  |
| C6.09 | 2 | COM | 2 | **Buddy** indicates readiness to move on |  |
| C6.10 | 2 | COM | 1 | **HCW** indicates readiness to move on |  |
| C6Addtl | 2 |  |  | **Additional TMWK Behaviors observed in Step 5** |  |
| **Step 7: HAND HYGIENE PERFORMANCE #2 (HCW only, in doffing room)** | | | | |  |
| C7.01 | 2 | COM | 1 | **HCW** signals (vbl and/or nvbl) understanding of TO instructions or asks for clarification. |  |
| C7.02 | 2 | SA | 1 | **HCW** ‘talks out’ the HH process |  |
| C7.03 | 2 | SA | 2 | **Buddy** watches HCW perform HH |  |
| C7.04 | 2 | SA | 3 | **TO** watches HCW perform HH |  |
| C7.05 | 2 | MS | 3 | **TO** offers reinforcing and/or corrective feedback on HCW’s HH |  |
| C7.06 | 2 | SA | 1 | **HCW** signals (vbl and/or nvbl) when complete |  |
| C7Addtl | 2 |  |  | **Additional TMWK Behaviors observed in Step 7** |  |
| **Step 8: OUTER GLOVE REMOVAL** | | |  |  |  |
| C8.01 | 2 | MS | 3 | **TO** verbally describes act of outer glove removal. |  |
| C8.02 | 2 | MS | 3 | **TO** mimes act of outer glove removal. |  |
| C8.03 | 2 | COM | 1 | **HCW** signals (vbl and/or nvbl) understanding of instructions or asks for clarification |  |
| C8.04 | 2 | SA | 1 | **HCW** ‘talks out’ the glove removal process |  |
| C8.05 | 2 | SA | 2 | **Buddy** watches HCW remove gloves |  |
| C8.06 | 2 | SA | 3 | **TO** watches HCW remove glove |  |
| C8.07 | 2 | MS | 3 | **TO** offers HCW reinforcing and/or corrective feedback on glove removal |  |
| C8.08 | 2 | SA | 1 | **HCW** signals (vbl and/or nvbl) when complete |  |
| C8.09 | 2 | SA | 3 | **TO** points out places of possible contamination to team or indicates that none is observed |  |
| C8.10 | 2 | SA | 1 | **HCW** points out places of possible contamination to team or indicates that none is observed |  |
| C8.11 | 2 | SA | 2 | **Buddy** points out places of possible contamination to team or indicates that none is observed |  |
| C8.12 | 2 | SA | 3 | **TO** asks team if they are ready to proceed to next step |  |
| C8.13 | 2 | COM | 2 | **Buddy** indicates readiness to move on |  |
| C8.14 | 2 | COM | 1 | **HCW** indicates readiness to move on |  |
| **Step 9: HAND HYGIENE PERFORMANCE #3 (HCW only)** | | | |  |  |
| C9.01 | 2 | COM | 1 | **HCW** signals (vbl and/or nvbl) understanding of TO instructions or asks for clarification. |  |
| C9.02 | 2 | SA | 1 | **HCW** ‘talks out’ the HH process |  |
| C9.03 | 2 | SA | 2 | **Buddy** watches HCW perform HH |  |
| C9.04 | 2 | SA | 3 | **TO** watches HCW perform HH |  |
| C9.05 | 2 | MS | 3 | **TO** offers reinforcing and/or corrective feedback on HCW’s HH |  |
| C9.06 | 2 | SA | 1 | **HCW** signals (vbl and/or nvbl) when complete |  |
| C9.07 | 2 | SA | 3 | **TO** asks team if they are ready to proceed to next step |  |
| C9.08 | 2 | COM | 2 | **Buddy** indicates readiness to move on |  |
| C9.09 | 2 | COM | 1 | **HCW** indicates readiness to move on |  |
| C9Addtl | 2 |  |  | **Additional TMWK Behaviors observed in Step 9** |  |
| **RISKY Step 10: PAPR REMOVAL** | | |  |  |  |
| **RISKY Step 10a: HOSE & BELT REMOVAL** | | | |  |  |
| C10.01 | 2 | MS | 3 | **TO** verbally describes PAPR hose removal |  |
| C10.02 | 2 | MS | 3 | **TO** mimes PAPR hose removal |  |
| C10.03 | 2 | SA | 3 | **TO or Buddy** warns HCW that they will not have airflow once hose is disconnected |  |
| C10.04 | 2 | COM | 1 | **HCW** signals (vbl and/or nvbl) understanding of instructions or asks for clarification |  |
| C10.05 | 2 | MS | 3 | **TO** and/or **Buddy** acknowledge the discomfort and risk HCW may experience but remind HCW they are a support network |  |
| C10.06 | 2 | COM | 2 | **Buddy** signals (vbl and/or nvbl) understanding of instructions or asks for clarification |  |
| C10.07 | 2 | SA | 2 | **Buddy** confirms with HCW readiness to remove PAPR hose |  |
| C10.08 | 2 | SA | 2 | **Buddy** ‘talks out’ process of disconnecting hose |  |
| C10.09 | 2 | SA | 3 | **TO** watches Buddy disconnect hose |  |
| C10.10 | 2 | MS | 3 | **TO** offers reinforcing and/or corrective feedback on hose removal |  |
| C10.11 | 2 | SA | 1 | **HCW** 'talks out' unsapping battery pack |  |
| C10.12 | 2 | SA | 2 | **Buddy** 'talks out' process of placig battery pack in bucket |  |
| C10.13 | 2 | MS | 3 | **TO** offers reinforcing and/or corrective feedback on battery pack removal |  |
| C10aAddtl | 2 |  |  | **Additional TMWK Behaviors observed in Step 10a** |  |
| **RISKY Step 10b: HOOD REMOVAL** | | |  |  |  |
| C10.14 | 2 | MS | 3 | **TO** verbally describes PAPR hood removal (might be combined in hose removal instruction) |  |
| C10.15 | 2 | MS | 3 | **TO** mimes PAPR hood removal (might be combined in hose removal instruction) |  |
| C10.16 | 2 | COM | 2 | **Buddy** signals (vbl and/or nvbl) understanding of instructions or asks for clarification |  |
| C10.17 | 2 | COM | 2 | **Buddy** confirms HCW readiness to roll up shroud |  |
| C10.18 | 2 | SA | 2 | **Buddy** 'talks out' process of rolling shroud up |  |
| C10.19 | 2 | MS | 3 | **TO** offers reinforcing and/or corrective feedback on shroud roll-up |  |
| C10.20 | 2 | COM | 1 | **HCW** signals (vbl and/or nvbl) understanding of instructions or asks for clarification |  |
| C10.21 | 2 | MS | 3 | **TO** and/or **Buddy** reassures HCW will be out of hood soon |  |
| C10.22 | 2 | SA | 1 | **HCW** ‘talks out’ PAPR removal |  |
| C10.23 | 2 | SA | 3 | **TO** watches HCW remove PAPR hood |  |
| C10.24 | 2 | SA | 2 | **Buddy** watches HCW remove PAPR hood |  |
| C10.25 | 2 | MS | 3 | **TO** offers reinforcing and/or corrective feedback on hood removal |  |
| C10.26 | 2 | SA | 1 | **HCW** points out places of possible contamination to team or indicates that none is observed |  |
| C10.27 | 2 | SA | 2 | **Buddy** points out places of possible contamination to team or indicates that none is observed |  |
| C10.28 | 2 | SA | 3 | **TO** points out places of possible contamination to team or indicates that none is observed |  |
| C10.29 | 2 | SA | 3 | **TO** asks team if they are ready to proceed to next step |  |
| C10.30 | 2 | COM | 2 | **Buddy** indicates readiness to move on |  |
| C10.31 | 2 | COM | 1 | **HCW** indicates readiness to move on |  |
| C10bAddtl | 2 |  |  | **Additional TMWK Behaviors observed in Step 10b** |  |
| **Step 11: HAND HYGIENE PERFORMANCE #4 (HCW & Buddy)** | | | | |  |
| C11.01 | 2 | COM | 2 | **Buddy** signals (vbl and/or nvbl) understanding of TO instructions or asks for clarification. |  |
| C11.02 | 2 | SA | 2 | **Buddy** ‘talks out’ the HH process |  |
| C11.03 | 2 | SA | 1 | **HCW** watches Buddy perform HH |  |
| C11.04 | 2 | SA | 3 | **TO** watches Buddy perform HH |  |
| C11.05 | 2 | MS | 3 | **TO** offers reinforcing and/or corrective feedback on Buddy’s HH |  |
| C11.06 | 2 | SA | 2 | **Buddy** signals (vbl and/or nvbl) when complete |  |
| C11.07 | 2 | COM | 1 | **HCW** signals (vbl and/or nvbl) understanding of TO instructions or asks for clarification. |  |
| C11.08 | 2 | SA | 1 | **HCW** ‘talks out’ the HH process |  |
| C11.09 | 2 | SA | 2 | **Buddy** watches HCW perform HH |  |
| C11.10 | 2 | SA | 3 | **TO** watches HCW perform HH |  |
| C11.11 | 2 | MS | 3 | **TO** offers reinforcing and/or corrective feedback on HCW’s HH |  |
| C11.12 | 2 | SA | 1 | **HCW** signals (vbl and/or nvbl) when complete |  |
| C11.13 | 2 | SA | 3 | **TO** asks team if they are ready to proceed to next step |  |
| C11.14 | 2 | COM | 2 | **Buddy** indicates readiness to move on |  |
| C11.15 | 2 | COM | 1 | **HCW** indicates readiness to move on |  |
| C11Addtl | 2 |  |  | **Additional TMWK Behaviors observed in Step 11** |  |
| **RISKY Step 12: SURGICAL GOWN REMOVAL** | | | |  |  |
| C12.01 | 2 | MS | 3 | **TO** verbally describes act of surgical gown removal |  |
| C12.02 | 2 | MS | 3 | **TO** mimes act of surgical gown removal |  |
| C12.03 | 2 | COM | 1 | **HCW** signals (vbl and/or nvbl) understanding of instructions or asks for clarification |  |
| C12.04 | 2 | SA | 1 | **HCW** ‘talks out’ the gown removal process |  |
| C12.05 | 2 | SA | 3 | **TO** watches HCW remove gown |  |
| C12.06 | 2 | SA | 2 | **Buddy** watches HCW remove gown |  |
| C12.07 | 2 | MS | 3 | **TO** offers reinforcing and/or corrective feedback on HCW’s gown removal technique |  |
| C12.08 | 2 | SA | 1 | **HCW** signals (vbl and/or nvbl) when complete |  |
| C12.09 | 2 | SA | 1 | **HCW** points out places of possible contamination to team or indicates that none is observed |  |
| C12.10 | 2 | SA | 2 | **Buddy** points out places of possible contamination to team or indicates that none is observed |  |
| C12.11 | 2 | SA | 3 | **TO** points out places of possible contamination to team or indicates that none is observed |  |
| C12.12 | 2 | SA | 3 | **TO** asks team if they are ready to proceed to next step |  |
| C12.13 | 2 | COM | 2 | **Buddy** indicates readiness to move on |  |
| C12.14 | 2 | COM | 1 | **HCW** indicates readiness to move on |  |
| C12Addtl | 2 |  |  | **Additional TMWK Behaviors observed in Step 12** |  |
| **Step 13: HAND HYGIENE PERFORMANCE #5 (HCW & Buddy)** | | | | |  |
| C13.01 | 2 | COM | 2 | **Buddy** signals (vbl and/or nvbl) understanding of TO instructions or asks for clarification. |  |
| C13.02 | 2 | SA | 2 | **Buddy** ‘talks out’ the HH process |  |
| C13.03 | 2 | SA | 1 | **HCW** watches Buddy perform HH |  |
| C13.04 | 2 | SA | 3 | **TO** watches Buddy perform HH |  |
| C13.05 | 2 | MS | 3 | **TO** offers reinforcing and/or corrective feedback on Buddy’s HH |  |
| C13.06 | 2 | SA | 2 | **Buddy** signals (vbl and/or nvbl) when complete |  |
| C13.07 | 2 | COM | 1 | **HCW** signals (vbl and/or nvbl) understanding of TO instructions or asks for clarification. |  |
| C13.08 | 2 | SA | 1 | **HCW** ‘talks out’ the HH process |  |
| C13.09 | 2 | SA | 2 | **Buddy** watches HCW perform HH |  |
| C13.10 | 2 | SA | 3 | **TO** watches HCW perform HH |  |
| C13.11 | 2 | MS | 3 | **TO** offers reinforcing and/or corrective feedback on HCW’s HH |  |
| C13.12 | 2 | SA | 1 | **HCW** signals (vbl and/or nvbl) when complete |  |
| C13.13 | 2 | SA | 3 | **TO** asks team if they are ready to proceed to next step |  |
| C13.14 | 2 | COM | 2 | **Buddy** indicates readiness to move on |  |
| C13.15 | 2 | COM | 1 | **HCW** indicates readiness to move on |  |
| **RISKY Step 14: BOOT COVER REMOVAL & SHOE DISENFECTION** | | | | |  |
| C14.01 | 2 | MS | 3 | **TO** verbally describes act of boot cover removal |  |
| C14.02 | 2 | MS | 3 | **TO** mimes act of boot cover removal |  |
| C14.03 | 2 | COM | 2 | **Buddy** signals (vbl and/or nvbl) understanding of instructions or asks for clarification |  |
| C14.04 | 2 | COM | 1 | **HCW** signals (vbl and/or nvbl) understanding of instructions or asks for clarification |  |
| C14.05 | 2 | SA | 2 | **Buddy** asks if HCW is ready to be assisted with boot cover removal |  |
| C14.06 | 2 | COM | 1 | **HCW** confirms readiness to remove boot cover |  |
| C14.07 | 2 | SA | 2 | **Buddy** ‘talks out’ boot removal process |  |
| C14.08 | 2 | SA | 3 | **TO** watches Buddy remove boot cover |  |
| C14.09 | 2 | SA | 2 | **Buddy** signals (vbl and/or nvbl) when complete |  |
| C14.10 | 2 | MS | 3 | **TO** offers reinforcing and/or corrective feedback to Buddy and/or HCW |  |
| C14.11 | 2 | SA | 1 | **HCW** signals (vbl and/or nvbl) when complete |  |
| C14.12 | 2 | SA | 1 | **HCW** points out places of possible contamination to team or indicates that none is observed |  |
| C14.13 | 2 | SA | 2 | **Buddy** points out places of possible contamination to team or indicates that none is observed |  |
| C14.14 | 2 | SA | 3 | **TO** points out places of possible contamination to team or indicates that none is observed |  |
| C14.15 | 2 | SA | 3 | **TO** asks team if they are ready to proceed to next step |  |
| C14.16 | 2 | COM | 2 | **Buddy** indicates readiness to move on |  |
| C14.17 | 2 | COM | 1 | **HCW** indicates readiness to move on |  |
| C14Addtl | 2 |  |  | **Additional TMWK Behaviors observed in Step 14** |  |
| **Step 15: HAND HYGIENE PERFORMANCE #6 (HCW & Buddy)** | | | | |  |
| C15.01 | 2 | COM | 2 | **Buddy** signals (vbl and/or nvbl) understanding of TO instructions or asks for clarification. |  |
| C15.02 | 2 | SA | 2 | **Buddy** ‘talks out’ the HH process |  |
| C15.03 | 2 | SA | 1 | **HCW** watches Buddy perform HH |  |
| C15.04 | 2 | SA | 3 | **TO** watches Buddy perform HH |  |
| C15.05 | 2 | MS | 3 | **TO** offers reinforcing and/or corrective feedback on Buddy’s HH |  |
| C15.06 | 2 | SA | 2 | **Buddy** signals (vbl and/or nvbl) when complete |  |
| C15.07 | 2 | COM | 1 | **HCW** signals (vbl and/or nvbl) understanding of TO instructions or asks for clarification. |  |
| C15.08 | 2 | SA | 1 | **HCW** ‘talks out’ the HH process |  |
| C15.09 | 2 | SA | 2 | **Buddy** watches HCW perform HH |  |
| C15.10 | 2 | SA | 3 | **TO** watches HCW perform HH |  |
| C15.11 | 2 | MS | 3 | **TO** offers reinforcing and/or corrective feedback on HCW’s HH |  |
| C15.12 | 2 | SA | 1 | **HCW** signals (vbl and/or nvbl) when complete |  |
| C15Addtl | 2 |  |  | **Additional TMWK Behaviors observed in Step 15** |  |
| **Step 16: INNER GLOVE REMOVAL** | | |  |  |  |
| C16.01 | 2 | MS | 3 | **TO** verbally describes act of glove removal |  |
| C16.02 | 2 | MS | 3 | **TO**  mimes act of glove removal |  |
| C16.03 | 2 | COM | 1 | **HCW** signals (vbl and/or nvbl) understanding of instructions or asks for clarification |  |
| C16.04 | 2 | SA | 1 | **HCW** ‘talks out’ the glove removal process |  |
| C16.05 | 2 | SA | 2 | **Buddy** watches HCW remove gloves |  |
| C16.06 | 2 | SA | 3 | **TO** watches HCW remove glove |  |
| C16.07 | 2 | MS | 3 | **TO** offers HCW reinforcing and/or corrective feedback on glove removal |  |
| C16.08 | 2 | SA | 1 | **HCW** signals (vbl and/or nvbl) when complete |  |
| C16.09 | 2 | SA | 3 | **TO** points out places of possible contamination to team or indicates that none is observed |  |
| C16.10 | 2 | SA | 1 | **HCW** points out places of possible contamination to team or indicates that none is observed |  |
| C16.11 | 2 | SA | 2 | **Buddy** points out places of possible contamination to team or indicates that none is observed |  |
| C16.12 | 2 | SA | 3 | **TO** asks team if they are ready to proceed to next step |  |
| C16.13 | 2 | COM | 2 | **Buddy** indicates readiness to move on |  |
| C16.14 | 2 | COM | 1 | **HCW** indicates readiness to move on |  |
| **Step 17: HAND HYGIENE PERFORMANCE #7 (HCW only)** | | | | |  |
| C17.01 | 2 | COM | 1 | **HCW** signals (vbl and/or nvbl) understanding of TO instructions or asks for clarification. |  |
| C17.02 | 2 | SA | 1 | **HCW** ‘talks out’ the HH process |  |
| C17.03 | 2 | SA | 2 | **Buddy** watches HCW perform HH |  |
| C17.04 | 2 | SA | 3 | **TO** watches HCW perform HH |  |
| C17.05 | 2 | MS | 3 | **TO** offers reinforcing and/or corrective feedback on HCW’s HH |  |
| C17.06 | 2 | SA | 1 | **HCW** signals (vbl and/or nvbl) when complete |  |
| **Step 18: ASSESS FOR VISIBLE CONTAMINATION #2** | | | |  |  |
| C18.01 | 2 | COM | 1 | **HCW** signals (vbl and/or nvbl) understanding of TO instructions or asks for clarification. |  |
| C18.02 | 2 | MS | 1 | **HCW** turns in circles to facilitate Buddy and TO assessment of PPE for contamination |  |
| C18.03 | 2 | SA | 1 | **HCW** points out places of possible contamination to team or indicates that none is observed |  |
| C18.04 | 2 | SA | 2 | **Buddy** points out places of possible contamination to team or indicates that none is observed |  |
| C18.05 | 2 | SA | 3 | **TO** points out places of possible contamination to team or indicates that none is observed |  |
| C18Addtl | 2 |  |  | **Additional TMWK Behaviors observed in Step 18** |  |

| **Table S2. Observation Tool: Treatment Order** | | | | | |
| --- | --- | --- | --- | --- | --- |
| **STEP** | **ROOM  1 = Patient 2 = Doffing** | **COMPTNCY** | **ACTOR 1 = HCW 2 = Buddy 3 = TO** | **BEHAVIOR** | **0 = N 1 = Y 2 = Uncertain 3 = NA OBSERVED** |
| **Step 1: PREPARATION FOR DOFFING (in patient room)** | | | | |  |
| T1.01 | 1 | SA | 3 | **TO** orients Buddy to doffing room layout |  |
| T1.02 | 1 | COM | 1 | **HCW** signals (vbl and/or nvbl) to engage TO and Buddy |  |
| T1.03 | 1 | COM | 3 | **TO** acknowledges HCW (vbl and/or nvbl) |  |
| T1Addtl | 1 |  |  | **Additional TMWK Behaviors observed in Step 1** |  |
| **Step 2: HAND HYGIENE PERFORMANCE #1 (in patient room)** | | | | |  |
| T2.01 | 1 | MS | 3 | **TO** verbally describes act of HH |  |
| T2.02 | 1 | MS | 3 | **TO** mimes act of HH |  |
| T2.03 | 1 | COM | 1 | **HCW** signals (vbl and/or nvbl) understanding of TO instructions or asks for clarification. |  |
| T2.04 | 1 | SA | 1 | **HCW** ‘talks out’ the HH process |  |
| T2.05 | 1 | SA | 2 | **Buddy** watches HCW perform HH |  |
| T2.06 | 1 | SA | 3 | **TO** watches HCW perform HH |  |
| T2.07 | 1 | MS | 3 | **TO** offers reinforcing and/or corrective feedback on HCW’s HH |  |
| T2.08 | 1 | SA | 1 | **HCW** signals (vbl and/or nvbl) when complete |  |
| **Step 3: Exam Glove Removal (in patient room)** | | | |  |  |
| T3.01 | 1 | MS | 3 | **TO** verbally describes act of outer glove removal. |  |
| T3.02 | 1 | MS | 3 | **TO** mimes act of outer glove removal. |  |
| T3.03 | 1 | COM | 1 | **HCW** signals (vbl and/or nvbl) understanding of instructions or asks for clarification |  |
| T3.04 | 1 | SA | 1 | **HCW** ‘talks out’ the glove removal process |  |
| T3.05 | 1 | SA | 2 | **Buddy** watches HCW remove gloves |  |
| T3.06 | 1 | SA | 3 | **TO** watches HCW remove glove |  |
| T3.07 | 1 | MS | 3 | **TO** offers HCW reinforcing and/or corrective feedback on glove removal |  |
| T3.08 | 1 | SA | 1 | **HCW** signals (vbl and/or nvbl) when complete |  |
| T3.09 | 1 | SA | 3 | **TO** points out places of possible contamination to team or indicates that none is observed |  |
| T3.10 | 1 | SA | 1 | **HCW** points out places of possible contamination to team or indicates that none is observed |  |
| T3.11 | 1 | SA | 2 | **Buddy** points out places of possible contamination to team or indicates that none is observed |  |
| T3.12 | 1 | SA | 3 | **TO** asks team if they are ready to proceed to next step |  |
| T3.13 | 1 | COM | 2 | **Buddy** indicates readiness to move on |  |
| T3.14 | 1 | COM | 1 | **HCW** indicates readiness to move on |  |
| T3Addtl | 1 |  |  | **Additional TMWK Behaviors observed in Step 3** |  |
| **Step 4: Hand Hygiene Performance # 2 (in patient room)** | | | | |  |
| T4.01 | 1 | COM | 1 | **HCW** signals (vbl and/or nvbl) understanding of TO instructions or asks for clarification. |  |
| T4.02 | 1 | SA | 1 | **HCW** ‘talks out’ the HH process |  |
| T4.03 | 1 | SA | 2 | **Buddy** watches HCW perform HH |  |
| T4.04 | 1 | SA | 3 | **TO** watches HCW perform HH |  |
| T4.05 | 1 | MS | 3 | **TO** offers reinforcing and/or corrective feedback on HCW’s HH |  |
| T4.06 | 1 | SA | 1 | **HCW** signals (vbl and/or nvbl) when complete |  |
|  | 1 |  |  | **Additional TMWK Behaviors observed in Step 4** |  |
| **Step 5: ASSESS HCW PPE FOR VISIBLE CONTAMINATION (in patient room)** | | | | |  |
| T5.01 | 1 | COM | 1 | **HCW** signals (vbl and/or nvbl) understanding of TO instructions or asks for clarification. |  |
| T5.02 | 1 | SA | 3 | **TO** visually inspects HCW for visible contamination. |  |
| T5.03 | 1 | SA | 2 | **Buddy** visually inspects HCW for visible contamination |  |
| T5.04 | 1 | MS | 1 | **HCW** turns in circles to facilitate Buddy and TO assessment of PPE for contamination |  |
| T5.05 | 1 | SA | 1 | **HCW** points out places of possible contamination to team or indicates that none is observed |  |
| T5.06 | 1 | SA | 2 | **Buddy** points out places of possible contamination to team or indicates that none is observed |  |
| T5.07 | 1 | SA | 3 | **TO** points out places of possible contamination to team or indicates that none is observed |  |
| T5.08 | 1 | SA | 3 | **TO** asks team if they are ready to proceed to next step |  |
| T5.09 | 1 | COM | 2 | **Buddy** indicates readiness to move on |  |
| T5.10 | 1 | COM | 1 | **HCW** indicates readiness to move on |  |
| T5Addtl | 1 |  |  | **Additional TMWK Behaviors observed in Step 3** |  |
| **Step 6: YELLOW ISOLATION GOWN REMOVAL (In patient room)** | | | | |  |
| T6.01 | 1 | MS | 3 | **TO** verbally describes act of isolation gown removal. |  |
| T6.02 | 1 | MS | 3 | **TO** mimes  act of isolation gown removal. |  |
| T6.03 | 1 | COM | 1 | **HCW** signals (vbl and/or nvbl) understanding of instructions or asks for clarification |  |
| T6.04 | 1 | SA | 1 | **HCW** ‘talks out’ the gown removal process |  |
| T6.05 | 1 | SA | 2 | **Buddy** watches HCW remove gown |  |
| T6.06 | 1 | SA | 3 | **TO** watches HCW remove gown |  |
| T6.07 | 1 | MS | 3 | **TO** offers HCW reinforcing and/or corrective feedback on gown removal |  |
| T6.08 | 1 | SA | 1 | **HCW** signals (vbl and/or nvbl) when complete |  |
| T6.09 | 1 | SA | 3 | **TO** asks team if they are ready to proceed to next step |  |
| T6.10 | 1 | COM | 2 | **Buddy** indicates readiness to move on |  |
| T6.11 | 1 | COM | 1 | **HCW** indicates readiness to move on |  |
| T6Addtl | 1 |  |  | **Additional TMWK Behaviors observed in Step 4** |  |
| **Step 7: HAND HYGIENE (#3)** | | |  |  |  |
| T7.01 | 1 | COM | 1 | **HCW** signals (vbl and/or nvbl) understanding of TO instructions or asks for clarification. |  |
| T7.02 | 1 | SA | 1 | **HCW** ‘talks out’ the HH process |  |
| T7.03 | 1 | SA | 2 | **Buddy** watches HCW perform HH |  |
| T7.04 | 1 | SA | 3 | **TO** watches HCW perform HH |  |
| T7.05 | 1 | MS | 3 | **TO** offers reinforcing and/or corrective feedback on HCW’s HH |  |
| T7.06 | 1 | SA | 1 | **HCW** signals (vbl and/or nvbl) when complete |  |
| T7.07 | 1 | SA | 3 | **TO** asks team if they are ready to exit room (proceed to next step) |  |
| T7.08 | 1 | COM | 1 | **HCW** indicates readiness to exit |  |
| T7.09 | 1 | COM | 2 | **Buddy** indicates readiness to exit |  |
| T7Addtl | 1 |  |  | **Additional TMWK Behaviors observed in Step 7** |  |
| **Step 8: EXITING PATIENT ROOM; ENTERING DOFFING ROOM** | | | | |  |
| T8.01 | 2 | MS | 3 | **TO** verbally describes act of entering doffing room |  |
| T8.02 | 2 | MS | 3 | **TO** mimes act of entry |  |
| T8.03 | 2 | SA | 3 | **TO** watches HCW enter doffing room |  |
| T8.04 | 2 | SA | 2 | **Buddy** watches HCW enter doffing room |  |
| T8.05 | 2 | SA | 3 | **TO** introduces self and Buddy to HCW |  |
| T8.06 | 2 | SA | 3 | **TO** describes roles and responsibilities of all team members |  |
| T8.07 | 2 | SA | 3 | **TO** orients HCW to the doffing room layout |  |
| T8.08 | 2 | SA | 3 | **TO** emphasizes importance of maintaining spatial distance from trashcan and other surfaces |  |
| T8.09 | 2 | MS | 3 | **TO** encourages all team members to speak up and/or stop the doffing process when they have a concern |  |
| T8.10 | 2 | COM | 1 | **HCW** signals (vbl and/or nvbl) agreement with rules |  |
| T8.11 | 2 | COM | 2 | **Buddy** signals (vbl and/or nvbl) agreement with rules |  |
| T8.12 | 2 | SA | 3 | **TO** points out places of possible contamination to team or indicates that none is observed |  |
| T8.13 | 2 | SA | 1 | **HCW** points out places of possible contamination to team or indicates that none is observed |  |
| T8.14 | 2 | SA | 2 | **Buddy** points out places of possible contamination to team or indicates that none is observed |  |
| T8.15 | 2 | SA | 3 | **TO** asks team if they are ready to proceed to next step |  |
| T8.16 | 2 | COM | 2 | **Buddy** indicates readiness to move on |  |
| T8.17 | 2 | COM | 1 | **HCW** indicates readiness to move on |  |
| T8Addtl | 2 |  |  | **Additional TMWK Behaviors observed in Step 8** |  |
| **Step 9: HAND HYGIENE #3** | | |  |  |  |
| T9.01 | 2 | COM | 2 | **Buddy** signals (vbl and/or nvbl) understanding of TO instructions or asks for clarification. |  |
| T9.02 | 2 | SA | 2 | **Buddy** ‘talks out’ the HH process |  |
| T9.03 | 2 | SA | 1 | **HCW** watches Buddy perform HH |  |
| T9.04 | 2 | SA | 3 | **TO** watches Buddy perform HH |  |
| T9.05 | 2 | MS | 3 | **TO** offers reinforcing and/or corrective feedback on Buddy’s HH |  |
| T9.06 | 2 | SA | 2 | **Buddy** signals (vbl and/or nvbl) when complete |  |
| T9.07 | 2 | COM | 1 | **HCW** signals (vbl and/or nvbl) understanding of TO instructions or asks for clarification. |  |
| T9.08 | 2 | SA | 1 | **HCW** ‘talks out’ the HH process |  |
| T9.09 | 2 | SA | 2 | **Buddy** watches HCW perform HH |  |
| T9.10 | 2 | SA | 3 | **TO** watches HCW perform HH |  |
| T9.11 | 2 | MS | 3 | **TO** offers reinforcing and/or corrective feedback on HCW’s HH |  |
| T9.12 | 2 | SA | 1 | **HCW** signals (vbl and/or nvbl) when complete |  |
| T9Addtl | 2 |  |  | **Additional TMWK Behaviors observed in Step 9** |  |
| **Step 10: ASSESS HCW PPE FOR VISIBLE CONTAMINATION (in DOFFING room)** | | | | |  |
| T10.01 | 2 | COM | 1 | **HCW** signals (vbl and/or nvbl) understanding of TO instructions or asks for clarification. |  |
| T10.02 | 2 | SA | 3 | **TO** visually inspects HCW for visible contamination. |  |
| T10.03 | 2 | SA | 2 | **Buddy** visually inspects HCW for visible contamination |  |
| T10.04 | 2 | MS | 1 | **HCW** turns in circles to facilitate Buddy and TO assessment of PPE for contamination |  |
| T10.05 | 2 | SA | 1 | **HCW** points out places of possible contamination to team or indicates that none is observed |  |
| T10.06 | 2 | SA | 2 | **Buddy** points out places of possible contamination to team or indicates that none is observed |  |
| T10.07 | 2 | SA | 3 | **TO** points out places of possible contamination to team or indicates that none is observed |  |
| T10.08 | 2 | SA | 3 | **TO** asks team if they are ready to proceed to next step |  |
| T10.09 | 2 | COM | 2 | **Buddy** indicates readiness to move on |  |
| T10.10 | 2 | COM | 1 | **HCW** indicates readiness to move on |  |
| T10Addtl | 2 |  |  | **Additional TMWK Behaviors observed in Step X** |  |
| **Step 11: TAPE REMOVAL (Intervention ONLY)** | | | |  |  |
| T11.01 | 2 | MS | 3 | **TO** verbally describes act of tape removal. |  |
| T11.02 | 2 | MS | 3 | **TO** mimes act of tape removal. |  |
| T11.03 | 2 | COM | 1 | **HCW** signals (vbl and/or nvbl) understanding of instructions or asks for clarification |  |
| T11.04 | 2 | SA | 1 | **HCW** ‘talks out’ the tape removal process |  |
| T11.05 | 2 | SA | 2 | **Buddy** watches HCW remove tape |  |
| T11.06 | 2 | SA | 3 | **TO** watches HCW remove tape |  |
| T11.07 | 2 | MS | 3 | **TO** offers HCW reinforcing and/or corrective feedback on tape removal |  |
| T11.08 | 2 | SA | 1 | **HCW** signals (vbl and/or nvbl) when complete |  |
| T11.09 | 2 | SA | 3 | **TO** points out places of possible contamination to team or indicates that none is observed |  |
| T11.10 | 2 | SA | 1 | **HCW** points out places of possible contamination to team or indicates that none is observed |  |
| T11.11 | 2 | SA | 2 | **Buddy** points out places of possible contamination to team or indicates that none is observed |  |
| T11.12 | 2 | SA | 3 | **TO** asks team if they are ready to proceed to next step |  |
| T11.13 | 2 | COM | 2 | **Buddy** indicates readiness to move on |  |
| T11.14 | 2 | COM | 1 | **HCW** indicates readiness to move on |  |
| T11Addtl | 2 |  |  | **Additional TMWK Behaviors observed in Step 10** |  |
| **Step 12: FOURTH PERFORMANCE OF HAND HYGIENE** | | | | |  |
| T12.01 | 2 | COM | 1 | **HCW** signals (vbl and/or nvbl) understanding of TO instructions or asks for clarification. |  |
| T12.02 | 2 | SA | 1 | **HCW** ‘talks out’ the HH process |  |
| T12.03 | 2 | SA | 2 | **Buddy** watches HCW perform HH |  |
| T12.04 | 2 | SA | 3 | **TO** watches HCW perform HH |  |
| T12.05 | 2 | MS | 3 | **TO** offers reinforcing and/or corrective feedback on HCW’s HH |  |
| T12.06 | 2 | SA | 1 | **HCW** signals (vbl and/or nvbl) when complete |  |
| T12.07 | 2 | SA | 3 | **TO** asks team if they are ready to proceed to next step |  |
| T12.08 | 2 | COM | 2 | **Buddy** indicates readiness to move on |  |
| T12.09 | 2 | COM | 1 | **HCW** indicates readiness to move on |  |
| T12Addtl | 2 |  |  | **Additional TMWK Behaviors observed in Step 11** |  |
| **Step 13: OUTER GLOVE REMOVAL** | | |  |  |  |
| T13.01 | 2 | MS | 3 | **TO** verbally describes act of glove removal |  |
| T13.02 | 2 | MS | 3 | **TO** mimes  act of glove removal |  |
| T13.03 | 2 | COM | 1 | **HCW** signals (vbl and/or nvbl) understanding of instructions or asks for clarification |  |
| T13.04 | 2 | SA | 1 | **HCW** ‘talks out’ the glove removal process |  |
| T13.05 | 2 | SA | 2 | **Buddy** watches HCW remove gloves |  |
| T13.06 | 2 | SA | 3 | **TO** watches HCW remove glove |  |
| T13.07 | 2 | MS | 3 | **TO** offers HCW reinforcing and/or corrective feedback on glove removal |  |
| T13.08 | 2 | SA | 1 | **HCW** signals (vbl and/or nvbl) when complete |  |
| T13.09 | 2 | SA | 3 | **TO** points out places of possible contamination to team or indicates that none is observed |  |
| T13.10 | 2 | SA | 1 | **HCW** points out places of possible contamination to team or indicates that none is observed |  |
| T13.11 | 2 | SA | 2 | **Buddy** points out places of possible contamination to team or indicates that none is observed |  |
| T13.12 | 2 | SA | 3 | **TO** asks team if they are ready to proceed to next step |  |
| T13.13 | 2 | COM | 2 | **Buddy** indicates readiness to move on |  |
| T13.14 | 2 | COM | 1 | **HCW** indicates readiness to move on |  |
| T13Addtl | 2 |  |  | **Additional TMWK Behaviors observed in Step 12** |  |
| **Step 14: FIFTH PERFORMANCE OF HAND HYGIENE** | | | | |  |
| T14.01 | 2 | COM | 1 | **HCW** signals (vbl and/or nvbl) understanding of TO instructions or asks for clarification. |  |
| T14.02 | 2 | SA | 1 | **HCW** ‘talks out’ the HH process |  |
| T14.03 | 2 | SA | 2 | **Buddy** watches HCW perform HH |  |
| T14.04 | 2 | SA | 3 | **TO** watches HCW perform HH |  |
| T14.05 | 2 | MS | 3 | **TO** offers reinforcing and/or corrective feedback on HCW’s HH |  |
| T14.06 | 2 | SA | 1 | **HCW** signals (vbl and/or nvbl) when complete |  |
| T14.07 | 2 | SA | 3 | **TO** asks team if they are ready to proceed to next step |  |
| T14.08 | 2 | COM | 2 | **Buddy** indicates readiness to move on |  |
| T14.09 | 2 | COM | 1 | **HCW** indicates readiness to move on |  |
| T14Addtl | 2 |  |  | **Additional TMWK Behaviors observed in Step 9** |  |
| **RISKY Step 15: PAPR REMOVAL** | | |  |  |  |
| **RISKY Step 15a: HOSE & BELT REMOVAL** | | | |  |  |
| T15.01 | 2 | MS | 3 | **TO** verbally describes PAPR hose removal |  |
| T15.02 | 2 | MS | 3 | **TO** mimes PAPR hose removal |  |
| T15.03 | 2 | SA | 3 | **TO or Buddy** warns HCW that they will not have airflow once hose is disconnected |  |
| T15.04 | 2 | COM | 1 | **HCW** signals (vbl and/or nvbl) understanding of instructions or asks for clarification |  |
| T15.05 | 2 | MS | 3 | **TO** and/or **Buddy** acknowledge the discomfort and risk HCW may experience but remind HCW they are a support network |  |
| T15.06 | 2 | COM | 2 | **Buddy** signals (vbl and/or nvbl) understanding of instructions or asks for clarification |  |
| T15.07 | 2 | SA | 2 | **Buddy** confirms with HCW readiness to remove PAPR hose |  |
| T15.08 | 2 | SA | 2 | **Buddy** ‘talks out’ process of disconnecting hose |  |
| T15.09 | 2 | SA | 3 | **TO** watches Buddy disconnect hose |  |
| T15.10 | 2 | MS | 3 | **TO** offers reinforcing and/or corrective feedback on hose removal |  |
| T15.11 | 2 | SA | 1 | **HCW** 'talks out' unsapping battery pack |  |
| T15.12 | 2 | SA | 2 | **Buddy** 'talks out' process of placig battery pack in bucket |  |
| T15aAddtl | 2 |  |  | **Additional TMWK Behaviors observed in Step 14a** |  |
| **Step 16: MID-PAPR PERFORMANCE OF HAND HYGIENE (Buddy & HCW)** | | | | |  |
| T16.01 | 2 | COM | 2 | **Buddy** signals (vbl and/or nvbl) understanding of TO instructions or asks for clarification. |  |
| T16.02 | 2 | SA | 2 | **Buddy** ‘talks out’ the HH process |  |
| T16.03 | 2 | SA | 1 | **HCW** watches Buddy perform HH |  |
| T16.04 | 2 | SA | 3 | **TO** watches Buddy perform HH |  |
| T16.05 | 2 | MS | 3 | **TO** offers reinforcing and/or corrective feedback on Buddy’s HH |  |
| T16.06 | 2 | SA | 2 | **Buddy** signals (vbl and/or nvbl) when complete |  |
| T16.07 | 2 | COM | 1 | **HCW** signals (vbl and/or nvbl) understanding of TO instructions or asks for clarification. |  |
| T16.08 | 2 | SA | 1 | **HCW** ‘talks out’ the HH process |  |
| T16.09 | 2 | SA | 2 | **Buddy** watches HCW perform HH |  |
| T16.10 | 2 | SA | 3 | **TO** watches HCW perform HH |  |
| T16.11 | 2 | MS | 3 | **TO** offers reinforcing and/or corrective feedback on HCW’s HH |  |
| T16.12 | 2 | SA | 1 | **HCW** signals (vbl and/or nvbl) when complete |  |
| T16.13 | 2 | SA | 3 | **TO** asks team if they are ready to proceed to next step |  |
| T16.14 | 2 | COM | 2 | **Buddy** indicates readiness to move on |  |
| T16.15 | 2 | COM | 1 | **HCW** indicates readiness to move on |  |
| T16Addtl | 2 |  |  | **Additional TMWK Behaviors observed in Step 15** |  |
| **RISKY Step 15b: HOOD REMOVAL** | | |  |  |  |
| T15.13 | 2 | MS | 3 | **TO** verbally describes PAPR hood removal (might be combined in hose removal instruction) |  |
| T15.14 | 2 | MS | 3 | **TO** mimes PAPR hood removal (might be combined in hose removal instruction) |  |
| T15.15 | 2 | COM | 1 | **HCW** signals (vbl and/or nvbl) understanding of instructions or asks for clarification |  |
| T15.16 | 2 | MS | 3 | **TO** and/or **Buddy** reassures HCW will be out of hood soon |  |
| T15.17 | 2 | SA | 1 | **HCW** ‘talks out’ PAPR removal |  |
| T15.18 | 2 | SA | 3 | **TO** watches HCW remove PAPR hood |  |
| T15.19 | 2 | SA | 2 | **Buddy** watches HCW remove PAPR hood |  |
| T15.20 | 2 | MS | 3 | **TO** offers reinforcing and/or corrective feedback on hood removal |  |
| T15.21 | 2 | SA | 1 | **HCW** points out places of possible contamination to team or indicates that none is observed |  |
| T15.22 | 2 | SA | 2 | **Buddy** points out places of possible contamination to team or indicates that none is observed |  |
| T15.23 | 2 | SA | 3 | **TO** points out places of possible contamination to team or indicates that none is observed |  |
| T15.24 | 2 | SA | 3 | **TO** asks team if they are ready to proceed to next step |  |
| T15.25 | 2 | COM | 2 | **Buddy** indicates readiness to move on |  |
| T15.26 | 2 | COM | 1 | **HCW** indicates readiness to move on |  |
| T15Addtl | 2 |  |  | **Additional TMWK Behaviors observed in Step 14b`** |  |
| **Step 17: POST-PAPR PERFORMANCE OF HAND HYGIENE (HCW Only)** | | | | |  |
| T17.01 | 2 | COM | 1 | **HCW** signals (vbl and/or nvbl) understanding of TO instructions or asks for clarification. |  |
| T17.02 | 2 | SA | 1 | **HCW** ‘talks out’ the HH process |  |
| T17.03 | 2 | SA | 2 | **Buddy** watches HCW perform HH |  |
| T17.04 | 2 | SA | 3 | **TO** watches HCW perform HH |  |
| T17.05 | 2 | MS | 3 | **TO** offers reinforcing and/or corrective feedback on HCW’s HH |  |
| T17.06 | 2 | SA | 1 | **HCW** signals (vbl and/or nvbl) when complete |  |
| T17.07 | 2 | SA | 3 | **TO** asks team if they are ready to proceed to next step |  |
| T17.08 | 2 | COM | 2 | **Buddy** indicates readiness to move on |  |
| T17.09 | 2 | COM | 1 | **HCW** indicates readiness to move on |  |
|  | 2 |  |  | **Additional TMWK Behaviors observed in Step 15** |  |
| **RISKY Step 18a: SURGICAL GOWN REMOVAL (BUDDY)** | | | | |  |
| T18.01 | 2 | MS | 3 | **TO** verbally describes act of surgical gown removal to **Buddy** |  |
| T18.02 | 2 | MS | 3 | **TO** mimes act of surgical gown removal **to Buddy** |  |
| T18.03 | 2 | COM | 2 | **Buddy** signals (vbl and/or nvbl) understanding of untying gown instructions or asks for clarification |  |
| T18.04 | 2 | COM | 2 | **Buddy** confirms HCW readiness to untie gown |  |
| T18.05 | 2 | SA | 2 | **Buddy** 'talks out' process of untying gown |  |
| T18.06 | 2 | MS | 3 | **TO** offers reinforcing and/or corrective feedback on untying gown |  |
| T18.07 | 2 | COM | 2 | **Buddy** signals (vbl and/or nvbl) understanding of folding gown back instructions or asks for clarification |  |
| T18.08 | 2 | COM | 2 | **Buddy** confirms HCW readiness to fold gown |  |
| T18.09 | 2 | SA | 2 | **Buddy** 'talks out' process of folding gown |  |
| T18.10 | 2 | MS | 3 | **TO** offers reinforcing and/or corrective feedback on folding gown |  |
| T18aAddtl | 2 |  |  | **Additional TMWK Behaviors observed in Step 16a** |  |
| **Step 19a: POST-SURGICAL GOWN HAND HYGIENE (BUDDY)** | | | | |  |
| T19.01 | 2 | COM | 2 | **Buddy** signals (vbl and/or nvbl) understanding of TO instructions or asks for clarification. |  |
| T19.02 | 2 | SA | 2 | **Buddy** ‘talks out’ the HH process |  |
| T19.03 | 2 | SA | 1 | **HCW** watches Buddy perform HH |  |
| T19.04 | 2 | SA | 3 | **TO** watches Buddy perform HH |  |
| T19.05 | 2 | MS | 3 | **TO** offers reinforcing and/or corrective feedback on Buddy’s HH |  |
| T19.06 | 2 | SA | 2 | **Buddy** signals (vbl and/or nvbl) when complete |  |
| T19aAddtl | 2 |  |  | **Additional TMWK Behaviors observed in Step 17a** |  |
| **RISKY Step 18b: SURGICAL GOWN REMOVAL (HCW)** | | | | |  |
| T18.11 | 2 | MS | 3 | **TO** verbally describes act of surgical gown removal to **HCW** |  |
| T18.12 | 2 | MS | 3 | **TO** mimes act of surgical gown removal **to HCW** |  |
| T18.13 | 2 | COM | 1 | **HCW** signals (vbl and/or nvbl) understanding of instructions or asks for clarification |  |
| T18.14 | 2 | SA | 1 | **HCW** ‘talks out’ the gown removal process |  |
| T18.15 | 2 | SA | 3 | **TO** watches HCW remove gown |  |
| T18.16 | 2 | SA | 2 | **Buddy** watches HCW remove gown |  |
| T18.17 | 2 | MS | 3 | **TO** offers reinforcing and/or corrective feedback on HCW’s gown removal technique |  |
| T18.18 | 2 | SA | 1 | **HCW** signals (vbl and/or nvbl) when complete |  |
| T18.19 | 2 | SA | 1 | **HCW** points out places of possible contamination to team or indicates that none is observed |  |
| T18.20 | 2 | SA | 2 | **Buddy** points out places of possible contamination to team or indicates that none is observed |  |
| T18.21 | 2 | SA | 3 | **TO** points out places of possible contamination to team or indicates that none is observed |  |
| T18.22 | 2 | SA | 3 | **TO** asks team if they are ready to proceed to next step |  |
| T18.23 | 2 | COM | 2 | **Buddy** indicates readiness to move on |  |
| T18.24 | 2 | COM | 1 | **HCW** indicates readiness to move on |  |
| T18bAddtl | 2 |  |  | **Additional TMWK Behaviors observed in Step 16** |  |
| **Step 19b: POST-SURGICAL GOWN HAND HYGIENE (HCW)** | | | | |  |
| T19.07 | 2 | COM | 1 | **HCW** signals (vbl and/or nvbl) understanding of TO instructions or asks for clarification. |  |
| T19.08 | 2 | SA | 1 | **HCW** ‘talks out’ the HH process |  |
| T19.09 | 2 | SA | 2 | **Buddy** watches HCW perform HH |  |
| T19.10 | 2 | SA | 3 | **TO** watches HCW perform HH |  |
| T19.11 | 2 | MS | 3 | **TO** offers reinforcing and/or corrective feedback on HCW’s HH |  |
| T19.12 | 2 | SA | 1 | **HCW** signals (vbl and/or nvbl) when complete |  |
| T19.13 | 2 | SA | 3 | **TO** asks team if they are ready to proceed to next step |  |
| T19.14 | 2 | COM | 2 | **Buddy** indicates readiness to move on |  |
| T19.15 | 2 | COM | 1 | **HCW** indicates readiness to move on |  |
| T19Addtl | 2 |  |  | **Additional TMWK Behaviors observed in Step 17b** |  |
| **Step 20: INNER GLOVE REMOVAL** | | |  |  |  |
| T20.01 | 2 | MS | 3 | **TO** verbally describes act of glove removal |  |
| T20.02 | 2 | MS | 3 | **TO**  mimes act of glove removal |  |
| T20.03 | 2 | COM | 1 | **HCW** signals (vbl and/or nvbl) understanding of instructions or asks for clarification |  |
| T20.04 | 2 | SA | 1 | **HCW** ‘talks out’ the glove removal process |  |
| T20.05 | 2 | SA | 2 | **Buddy** watches HCW remove gloves |  |
| T20.06 | 2 | SA | 3 | **TO** watches HCW remove glove |  |
| T20.07 | 2 | MS | 3 | **TO** offers HCW reinforcing and/or corrective feedback on glove removal |  |
| T20.08 | 2 | SA | 1 | **HCW** signals (vbl and/or nvbl) when complete |  |
| T20.09 | 2 | SA | 3 | **TO** points out places of possible contamination to team or indicates that none is observed |  |
| T20.10 | 2 | SA | 1 | **HCW** points out places of possible contamination to team or indicates that none is observed |  |
| T20.11 | 2 | SA | 2 | **Buddy** points out places of possible contamination to team or indicates that none is observed |  |
| T20.12 | 2 | SA | 3 | **TO** asks team if they are ready to proceed to next step |  |
| T20.13 | 2 | COM | 2 | **Buddy** indicates readiness to move on |  |
| T20.14 | 2 | COM | 1 | **HCW** indicates readiness to move on |  |
| T20Addtl | 2 |  |  | **Additional TMWK Behaviors observed in Step 18** |  |
| **Step 21: EIGTH PERFORMANCE OF HAND HYGIENE** | | | | |  |
| T21.01 | 2 | COM | 1 | **HCW** signals (vbl and/or nvbl) understanding of TO instructions or asks for clarification. |  |
| T21.02 | 2 | SA | 1 | **HCW** ‘talks out’ the HH process |  |
| T21.03 | 2 | SA | 2 | **Buddy** watches HCW perform HH |  |
| T21.04 | 2 | SA | 3 | **TO** watches HCW perform HH |  |
| T21.05 | 2 | MS | 3 | **TO** offers reinforcing and/or corrective feedback on HCW’s HH |  |
| T21.06 | 2 | SA | 1 | **HCW** signals (vbl and/or nvbl) when complete |  |
| T21Addtl | 2 |  |  | **Additional TMWK Behaviors observed in Step 19** |  |
| **RISKY Step 22: BOOT COVER REMOVAL** | | | |  |  |
| T22.01 | 2 | MS | 3 | **TO** verbally describes act of boot cover removal |  |
| T22.02 | 2 | MS | 3 | **TO** mimes act of boot cover removal |  |
| T22.03 | 2 | COM | 2 | **Buddy** signals (vbl and/or nvbl) understanding of instructions or asks for clarification |  |
| T22.04 | 2 | SA | 2 | **Buddy** asks if HCW is ready to be assisted with boot cover removal |  |
| T22.05 | 2 | COM | 1 | **HCW** confirms readiness to remove boot cover |  |
| T22.06 | 2 | SA | 2 | **Buddy** ‘talks out’ boot removal process |  |
| T22.07 | 2 | SA | 3 | **TO** watches Buddy remove boot cover |  |
| T22.08 | 2 | MS | 3 | **TO** offers reinforcing and/or corrective feedback to Buddy and/or HCW |  |
| T22.09 | 2 | MS | 3 | **Buddy** or **TO** instructs HCW to adjust body for comfort while keeping to appropriate clean and dirty sides of the room |  |
| T22.10 | 2 | SA | 1 | **HCW** points out places of possible contamination to team or indicates that none is observed |  |
| T22.11 | 2 | SA | 2 | **Buddy** points out places of possible contamination to team or indicates that none is observed |  |
| T22.12 | 2 | SA | 3 | **TO** points out places of possible contamination to team or indicates that none is observed |  |
| T22.13 | 2 | SA | 3 | **TO** asks team if they are ready to proceed to next step |  |
| T22.14 | 2 | COM | 2 | **Buddy** indicates readiness to move on |  |
| T22.15 | 2 | COM | 1 | **HCW** indicates readiness to move on |  |
| T22Addtl | 2 |  |  | **Additional TMWK Behaviors observed in Step 20** |  |
| **Step 23: POST-BOOTCOVER HAND HYGIENE** | | | |  |  |
| T23.01 | 2 | COM | 2 | **Buddy** signals (vbl and/or nvbl) understanding of TO instructions or asks for clarification. |  |
| T23.02 | 2 | SA | 2 | **Buddy** ‘talks out’ the HH process |  |
| T23.03 | 2 | SA | 1 | **HCW** watches Buddy perform HH |  |
| T23.04 | 2 | SA | 3 | **TO** watches Buddy perform HH |  |
| T23.05 | 2 | MS | 3 | **TO** offers reinforcing and/or corrective feedback on Buddy’s HH |  |
| T23.06 | 2 | SA | 2 | **Buddy** signals (vbl and/or nvbl) when complete |  |
| T23.07 | 2 | COM | 1 | **HCW** signals (vbl and/or nvbl) understanding of TO instructions or asks for clarification. |  |
| T23.08 | 2 | SA | 1 | **HCW** ‘talks out’ the HH process |  |
| T23.09 | 2 | SA | 2 | **Buddy** watches HCW perform HH |  |
| T23.10 | 2 | SA | 3 | **TO** watches HCW perform HH |  |
| T23.11 | 2 | MS | 3 | **TO** offers reinforcing and/or corrective feedback on HCW’s HH |  |
| T23.12 | 2 | SA | 1 | **HCW** signals (vbl and/or nvbl) when complete |  |
| **Step 24: BOUFFANT REMOVAL** | | |  |  |  |
| T24.01 | 2 | MS | 3 | **TO** verbally describes act of bouffant removal |  |
| T24.02 | 2 | MS | 3 | **TO** mimes act of bouffant removal |  |
| T24.03 | 2 | COM | 1 | **HCW** signals (vbl and/or nvbl) understanding of instructions or asks for clarification |  |
| T24.04 | 2 | SA | 1 | **HCW** ‘talks out’ bouffant removal |  |
| T24.05 | 2 | SA | 2 | **Buddy** watches HCW remove bouffant |  |
| T24.06 | 2 | SA | 3 | **TO** watches HCW remove bouffant |  |
| T24.07 | 2 | MS | 3 | **TO** offers reinforcing and/or corrective feedback on HCW’s bouffant removal |  |
| T24.08 | 2 | SA | 1 | **HCW** signals (vbl and/or nvbl) when complete |  |
| T24Addtl | 2 |  |  | **Additional TMWK Behaviors observed in Step 22** |  |
| **Step 25: TENTH PERFORMANCE OF HAND HYGIENE** | | | | |  |
| T25.01 | 2 | COM | 1 | **HCW** signals (vbl and/or nvbl) understanding of TO instructions or asks for clarification. |  |
| T25.02 | 2 | SA | 1 | **HCW** ‘talks out’ the HH process |  |
| T25.03 | 2 | SA | 2 | **Buddy** watches HCW perform HH |  |
| T25.04 | 2 | SA | 3 | **TO** watches HCW perform HH |  |
| T25.05 | 2 | MS | 3 | **TO** offers reinforcing and/or corrective feedback on HCW’s HH |  |
| T25.06 | 2 | SA | 1 | **HCW** signals (vbl and/or nvbl) when complete |  |
| T25Addtl | 2 |  |  | **Additional TMWK Behaviors observed in Step 23** |  |
